# LobePrior segments lung lobes on computed tomography images in the presence of severe abnormalities

**DOI:** 10.1038/s41598-026-48136-8

**Published:** 2026-04-10

**Authors:** Jean Antonio Ribeiro, Diedre Santos do Carmo, Fabiano Reis, Ricardo Siufi Magalhães, Sergio San Juan Dertkigil, Simone Appenzeller, Letícia Rittner

**Affiliations:** 1 Universidade Estadual de Campinas, School of Electrical and Computer Engineering, Campinas, SP 13083-970 Brazil; 2 Universidade Estadual de Campinas, School of Medical Sciences, Campinas, SP 13083-970 Brazil; 3 Faculdade São Leopoldo Mandic, Department of Pulmonology, Campinas, SP 13045-755 Brazil

**Keywords:** CT Image, Deep Learning, Lung Lobe Segmentation, Medical Image Registration, Prior Information, Computed tomography, Machine learning

## Abstract

The development of robust algorithms for lung and lobe segmentation is essential for diagnosing and monitoring pulmonary diseases. Obtaining manual or automatic annotations is challenging, especially in patients with severe abnormalities due to poorly visible lobar fissures. We present LobePrior, an automated lung lobe segmentation method combining deep neural networks and probabilistic models. Segmentation occurs in three stages: a coarse stage processing downsampled images, a high-resolution stage where specialized AttUNets segment each lobe, and a final post-processing stage. Probabilistic models derived from label fusion guide the network in regions with severe abnormalities, and synthetic lesion generation provides augmentation during training. Performance was evaluated on LOLA11 and three additional datasets with cancerous nodules or COVID-19 consolidations. LobePrior achieved accurate segmentations compared to manual ground truth, reaching state-of-the-art performance even in challenging cases. On the LOCCA dataset, it obtained a Dice score of 0.966, with similar improvements on a COVID-19 CT dataset (Dice 0.978). Statistically significant improvements over competing methods were observed across all datasets. These results demonstrate that LobePrior effectively integrates anatomical priors and deep learning to provide reliable lobe segmentation in the presence of severe pulmonary abnormalities.

## Introduction

**Fig. 1:**
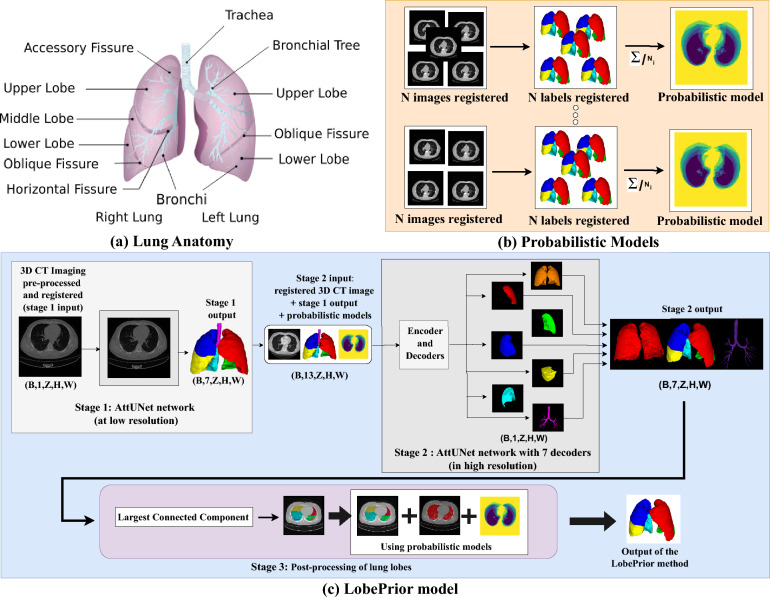
Overview of LobePrior method: lung anatomy, probabilistic models, and three-stage segmentation. **(a)** Image of a lung anatomy (adapted from Betts et al. ^38^), originally published in Anatomy and Physiology 2e (OpenStax), licensed under CC BY 4.0. **(b)** Diagram of the construction of probabilistic models, where $N_i$ is the number of images in group *i*. The images and annotations were grouped and registered to build probabilistic models. Accordingly, seven groups of probabilistic models were constructed. **(c)** Illustration of the LobePrior method, composed of three stages: first, low-resolution segmentation of lung lobes and airways; second, high-resolution refinement using multiple decoders, including airway (purple) and whole-lung (orange) optimization; and third, post-processing. In the figure, the letter *B* represents the *batch*, *Z* is *depth*, *H* is *height*, and *W* is the *width* of the CT image.

The pulmonary lobes are anatomical subdivisions of the lungs, delineated by pulmonary fissures (Fig. 1-a)). These structures enable the functional and structural separation of the lungs, facilitating ventilation and gas exchange. They also allow blood circulation through the pulmonary capillaries, where oxygenation occurs. The right lung consists of three lobes: superior, middle, and inferior. The left lung, due to the presence of the heart, is smaller and has two lobes: superior and inferior.

Pulmonary lobe segmentation enables quantification of disease involvement and helps characterize conditions such as pulmonary fibrosis, Chronic Obstructive Pulmonary Disease (COPD), lung cancer, and pneumonia, many of which predominantly affect specific lobes or regions of the lungs ^1^. Cancer, cystic fibrosis, and tuberculosis often affect the upper lobes, whereas interstitial pneumonia and panlobular emphysema frequently involve the lower lobes ^1^. The location of lung findings can be related to physiological processes. For example, COVID-19 most commonly affects the lower lobes of the lungs ^2^ because this region has greater blood perfusion and ventilation, making it more susceptible to the inflammatory involvement caused by the virus ^2^. In severe cases, the infection may extend to multiple lobes, resulting in a diffuse viral pneumonia pattern. In addition to the localization of abnormal findings, lung lobe segmentation can enable more precise planning for pulmonary surgeries (such as lobectomies), radiotherapy, and other interventions ^3^.

Manual identification of pulmonary fissures is essential for lobe segmentation, but it is time-consuming and labor-intensive, particularly in high-resolution CT scans ^1^. Specialists must analyze fissures slice by slice and annotate lobes using tools such as 3D Slicer ^4^ or ITK-SNAP ^5^. Because lobar fissures are often partially invisible or incomplete, radiologists typically infer lobe boundaries based on airway and vessel anatomy ^6^. Variations in fissure thickness, position, and shape due to respiratory and cardiac motion ^7^, as well as accessory fissures or fissures associated with the azygos vein ^8^, further complicate delineation. Pathological changes, nodules, or other fissure-like structures add additional ambiguity. Nevertheless, anatomical and pathological correlations make lobe boundary estimation possible even when fissures are incomplete ^9^.

Despite progress in automatic segmentation, there is still no robust method capable of handling severe opacifications commonly found in clinical CT scans. Resulting masks often contain holes, noise, or mislabelled voxels ^10^. Furthermore, no prior approach has combined convolutional neural networks (CNNs) with probabilistic models derived from registered CT images. The construction of such probabilistic representations remains challenging due to anatomical variability and respiratory deformations, which require accurate non-rigid registration, a computationally expensive and artifact-sensitive process ^11^. The effectiveness of these models also depends on large and diverse annotated datasets, including cases with severe abnormalities.

Given that pulmonary CT registration is feasible ^11,12^, incorporating prior anatomical information through probabilistic representations emerges as a promising strategy to guide neural networks in identifying missing or incomplete fissures. This study presents LobePrior, a fully automated method based on a multi-stage framework that seamlessly integrates convolutional neural networks (CNNs) with probabilistic priors for pulmonary lobe segmentation. Unlike conventional single-stage CNNs or purely atlas-based methods, LobePrior leverages anatomical priors to guide segmentation, particularly in regions with incomplete or invisible lobar fissures ^13,14^. This integration allows more accurate and robust lung lobe delineation in challenging cases with severe pulmonary abnormalities, distinguishing LobePrior from existing approaches. The model was trained on an enriched dataset containing new manually annotated cases with severe pulmonary abnormalities ^15^, as well as synthetic samples generated through controlled lesion insertion ^16^. Incorporating synthetic lesions exposes the network to a wider range of challenging scenarios, including consolidations that obscure lobar fissures, thereby enhancing both performance and generalization. In post-processing, probabilistic information refines low-confidence regions, correcting gaps caused by consolidations and nodules. This combination yields higher accuracy and generalization capability in real clinical scenarios, outperforming traditional approaches.

The contributions of this work can be summarized as follows:

- **Abnormal Lung Lobe Segmentation** - The main contribution of this work is the development of a novel approach for lung lobe segmentation in CT scans of patients with severe pulmonary diseases. By addressing the challenges posed by incomplete or obscured fissures, this method enhances the accuracy and reliability of automated lobe delineation.
- **Novel Hybrid Architecture** - The proposed method, named LobePrior, combines convolutional neural networks (CNNs) with probabilistic models. By incorporating prior information directly into the architecture, common results of low confidence lobe segmentation such as holes and undersegmentation are avoided.
- **Synthetic Lesion Insertion** - To mitigate overfitting and increase variability in the training set, a data augmentation strategy based on the insertion of synthetic lesions into CT images was developed.
- **Performance Evaluation** - The proposed method was compared with several approaches from the literature using multiple metrics, demonstrating statistically superior performance. Experiments involved a wide variety of volumetric CT scans from different sources. Accordingly, LobePrior was tested on four datasets, one with lung cancer cases and three with COVID-19 cases. The method was also evaluated based on the degree of impairment present in the CT volumes.
- **Availability and Reproducibility** - The source code is publicly available on GitHub (https://github.com/MICLab-Unicamp/LobePrior) and can be easily executed in a Python environment. The method has also been integrated into the MEDPSeg tool, developed by the MICLab group ^17^, ensuring reproducibility and ease of use by the scientific community.

## Related work

Automatic lung lobe segmentation in computed tomography (CT) images has been extensively investigated in the literature, with approaches evolving from classical model-based methods to more recent deep learning–based techniques. Early studies mainly explored atlas-based strategies, deformable registration, and statistical shape models, as well as explicit fissure detection using gradient-based filters, geometric analysis, or surface extraction. Although these methods achieved satisfactory results in scans with preserved anatomy, their performance is strongly dependent on fissure visibility and tends to degrade significantly in the presence of severe pathological changes, anatomical deformations, or imaging artifacts ^10^.

With the advancement of convolutional neural networks, deep learning–based methods have come to dominate the state of the art. Single-stage approaches, commonly based on U-Net, V-Net, and their 2D, 2.5D, or 3D variants, perform direct lobe segmentation from the input image ^1^. These methods typically report high Dice coefficients on datasets composed predominantly of normal cases; however, they still struggle to accurately delineate interlobar boundaries when fissures are incomplete, poorly contrasted, or disrupted by extensive lesions. As a result, considerable performance variability is observed across different lobes, particularly in the middle and lower lobes ^1^.

To mitigate these limitations, several studies have proposed multi-stage architectures or cascaded pipelines, in which global lung segmentation, fissure detection, and lobe segmentation are performed sequentially or iteratively ^18^. In general, these approaches achieve improvements in overlap- and surface-based metrics compared to single-stage models, at the cost of increased computational complexity and reliance on multiple independently trained modules. Nevertheless, their robustness remains limited in clinical scenarios characterized by severe pulmonary abnormalities, where fissures may be partially or entirely absent ^10^.

More recently, some studies have incorporated explicit anatomical or geometric knowledge into the segmentation process by using anatomical priors, graphs, surface-based models, or probabilistic formulations to regularize neural network predictions ^1^. These strategies aim to enforce anatomical consistency and reduce ambiguities in interlobar regions. Despite the reported advances, most of these approaches still evaluate performance primarily using the mean Dice coefficient, often on limited datasets that are not fully representative of complex pathological cases, with less emphasis on surface distance metrics and detailed statistical analyses.

The use of a priori information to handle anatomical variability has been successfully demonstrated for several organs ^13,14^, including the heart, thoracic structures, liver, and kidneys. To address inter-subject variability and mitigate the effects of potentially inaccurate anatomical information in fissure detection, van Rikxoort et al. ^12^ proposed an automatic lung lobe segmentation method that combines information from three anatomical structures: interlobar fissures, airways, and lung boundaries. First, a probabilistic lobe atlas is registered to the patient’s CT volume to provide an initial estimate of lobe locations. Subsequently, fissures are detected using dedicated filters, while airways and lung contours are extracted through specialized segmentation processes. These sources of evidence are integrated into a graph-cuts–based optimization framework that computes the final lobe segmentation by maximizing consistency with detected fissures and with anatomical constraints imposed by the airways and lung morphology. This combination enables accurate lobe segmentation even when fissures are absent or incomplete.

Among the most relevant works, Hofmanninger et al. ^19^, through the LungMask method, emphasized that the main challenges in automatic lung segmentation are not primarily related to model choice, but rather to the variability and diversity of the training data. The authors demonstrated that deep neural network–based segmentation models can achieve high accuracy when trained on sufficiently diverse datasets encompassing different populations, acquisition protocols, and pathologies. Their study highlights that increasing data variability improves model generalization, reducing failures in real clinical scenarios, and suggests that future advances in lung segmentation should prioritize the curation of more representative datasets.

Following a similar philosophy, Isensee et al. ^20^ introduced nnU-Net, a framework capable of self-configuring for a given task, including preprocessing, network architecture, training, and post-processing steps. This design makes it applicable to a wide range of new tasks without extensive manual tuning, with a strong focus on data processing and variability. Wasserthal et al. ^21^ further reinforced the importance of generalization by introducing TotalSegmentor, a 3D CNN for the automatic segmentation of 104 anatomical structures in CT images. The model was trained on a large and heterogeneous dataset, ensuring robustness and generalization across different CT acquisition scenarios. To improve the segmentation of small and complex structures, the method adopts a cascaded pipeline, in which larger structures are segmented first, followed by refinement of smaller and more challenging regions.

Although several techniques have reported satisfactory performance for lung lobe segmentation in CT images of healthy subjects, many of these methods were trained on datasets that include different pathologies without detailed characterization of the conditions considered or the number of cases per pathology ^10^. Moreover, there is a significant scarcity of images containing severe pulmonary lesions. Consequently, automatic lung lobe segmentation in CT scans affected by extensive opacities remains an open challenge.

Despite these advances, the literature indicates that while state-of-the-art methods achieve high performance under ideal conditions, important gaps remain regarding generalization and robustness in scans with incomplete fissures and severe pulmonary abnormalities ^10^. In this context, there is a clear need for approaches that combine the representational power of deep neural networks with explicit anatomical and probabilistic modeling, as well as comprehensive quantitative evaluations based on multiple performance metrics, in order to more faithfully reflect the challenges encountered in clinical practice.

## Materials and methods

In this section, we present the data, methodology, and experimental settings adopted in this study. First, we describe the datasets used and the incorporation of anatomical prior information through probabilistic models that capture structural variability and partial fissure visibility. Next, we detail the proposed method, LobePrior, which integrates these priors into a deep segmentation architecture. To enhance robustness against pathological variability, we employed synthetic lesion insertion and additional data augmentation strategies. Finally, we define the segmentation evaluation metrics and present the training protocol, including hyperparameters and the computational environment.

### Datasets

During the training phase of the network, we used a subset of the public LUNA16 ^22^ dataset, consisting of 50 computed tomography images with manual annotations provided by Tang et al. ^23^, including masks for the five pulmonary lobes, trachea, and lobar bronchi. Additionally, we created in-house annotations for the lung lobes on 100 images from various sources. The images were randomly selected from different public databases, aiming to compose a training set with wide anatomical and acquisition diversity, which enhances the model’s robustness and improves its convergence. This strategy also minimizes potential biases, ensuring that the dataset represents the variability observed in clinical images. In total, 150 volumetric images were used for the network training. For evaluation, three test datasets were used in the experiments, totaling 85 CT volumes: 60 from LOCCA ^24^, 15 from CT Images in COVID-19 ^25^, and 10 from CoronaCases ^15^. These datasets were completely excluded from the training phase (Table 1), ensuring an unbiased and representative validation of the method’s ability to handle different domains.

**Table 1:** Description of the manually annotated data sources, including pathology, the number of available images in each dataset, their respective amounts of manual annotations, mean and standard deviation (mean±std) of the number of axial slices per volume, and voxel size (mean±std). Only the dataset by Tang et al. ^23^ provides available annotations for the pulmonary lobes; in the other datasets, manual annotations were performed by the authors. The term “NOS” denotes that the dataset includes CT images from patients with either healthy lungs or lungs exhibiting non-specified pathological conditions.

Dataset Name	Pathology	Number of	Manual	Train	Test	Slices/Scan	Voxel Size
		Images	Annotations				($\textrm{mm}^{3}$)
**Datasets used exclusively for Training**							
ATM ^26^	NOS	399	in-house	10	-	663±140	0.33±0.19
COVID-CT-MD ^27^	COVID-19	305	in-house	25	-	108±195	2.72±0.28
COVID-LDCT ^28^	COVID-19	260	in-house	30	-	146±140	2.70±0.26
Luna16 (LIDC-IDRI) ^22^	cancer	888	in-house	15	-	256±134	0.78±0.44
LungCT-Diagnosis ^29^	COVID-19	61	in-house	5	-	24±150	15.10±5.02
MOSMED ^30^	COVID-19	1110	in-house	1	-	42±400	4.41±1.00
MSC ^31^	COVID-19	9	in-house	1	-	92±116	2.44±0.76
SM Mostafavi Dataverse ^32^	COVID-19	1013	in-house	3	-	802±185	0.20±0.05
Tang et al. (LIDC-IDRI) ^23^	cancer	888	public	50	-	256±134	0.78±0.44
**Datasets used for Training and Testing**							
CT Images in COVID-19 ^25^	COVID-19	771	in-house	10	15	71±460	2.98±0.89
**Datasets used exclusively for Testing**							
CoronaCases ^15^	COVID-19	20	in-house	-	10	258±390	0.59±0.15
LOCCA ^24^	COVID-19 and cancer	60	public	-	60	280±110	0.83±0.30
**Total**				150	85		

The internal annotations were performed by a trainee and subsequently verified by two radiologists with clinical experience in thoracic assessment, each with over 10 years of practice in the field. Each volume was individually analyzed based on the identification of anatomical fissures visible in the axial, coronal, and sagittal views of the computed tomography scans ^24^. In cases of uncertainty or incomplete fissures, the specialists relied on adjacent anatomical references, such as pulmonary vessels and bronchioles, to infer the lobar boundaries ^24^.

All CT images have manual voxel-level annotations for the pulmonary lobes and automatic annotations for the airways, generated by the MEDPSeg framework ^17^. The entire collection of images was combined to form a comprehensive dataset for network training, representing annotations at the lobar level, which were randomly divided into training and validation sets in an 80/20 percent ratio. In addition, the training, validation and test sets were defined to be mutually exclusive at the patient level, ensuring the absence of overlap between individuals. The combined dataset is highly heterogeneous, encompassing a wide variety of voxel sizes, manufacturers, acquisition parameters, and subtle differences in the annotation protocol. Some of these data sources were stored in the DICOM format, containing multiple series and modalities. Subsequently, these images were anonymized and converted to the NIfTI format ^33^.

Due to the heterogeneous origin of the datasets used in this study, there is a clear domain shift among them, related to differences in anatomical characteristics, acquisition protocols, and image resolution. To mitigate this effect, we adopted a random selection of images with broad diversity during training, in order to promote model generalization. Additionally, we worked with a limited number of manually segmented images, since this process is extremely labor-intensive and requires specialized expertise ^24^.

The training and evaluation of LobePrior were conducted using highly diverse datasets encompassing a wide range of pulmonary pathologies and CT acquisition conditions. The training data included manually annotated cases with severe abnormalities, complemented by synthetic lesions to simulate challenging clinical scenarios. The evaluation datasets comprised both standard cases and cases with consolidations, nodules, and incomplete lobar fissures. This broad diversity in pathologies, disease severity, and imaging protocols ensures that LobePrior is robust, generalizable, and capable of providing accurate segmentation across a wide spectrum of real-world clinical scenarios, highlighting its applicability in complex cases.

A standardized preprocessing pipeline was also applied, including intensity normalization and spatial registration of the volumes, to reduce inter-domain variability. For training and inference, all CT images were resampled to an isotropic voxel size of 1 mm^3^, their Hounsfield Unit (HU) values clipped to the range [-1024, 600], and subsequently normalized to the [0,1] range. Within this HU range, relevant information from the lungs and lobar fissures is preserved. To achieve robustness to such variations, the network architecture and training process incorporated appropriate regularization and optimization techniques.

### Incorporating prior information through probabilistic models

Inspired by Huang et al. ^34^ and Ma et al. ^14^, who incorporate probabilistic atlases as adaptive loss functions to inject anatomical knowledge into segmentation networks and improve performance in data-limited scenarios, we propose incorporating prior anatomical information through probabilistic models that encode the likelihood of lung lobe presence. These models are used to resolve ambiguities arising from low-confidence predictions, particularly in regions affected by severe abnormalities where fissures are partially or entirely obscured.

To construct such priors, we rely on co-registered CT images and their corresponding manual annotations from the public dataset of Tang et al. ^23^. Given the substantial anatomical variability observed in chest CT scans, multiple probabilistic templates are generated by grouping anatomically similar images and their labels ^12^. For each group, voxel-wise averaging of the registered binary labels yields a probabilistic representation of lobe presence.

The probabilistic models were generated from 50 volumetric CT images (Fig. 1-b). To capture anatomical and acquisition-related variability, the images and their corresponding labels were grouped according to structural similarity. First, all pre-processed volumes were mutually aligned using non-rigid registration to ensure spatial correspondence. Pairwise Dice similarity coefficients were then computed between the registered label maps. Volumes with Dice scores above 80 were grouped together, forming clusters of anatomically similar cases (Supplementary Algorithm 1).

For each group *g*, an initial probabilistic model $\tilde{\textbf{P}}^{(g)} = \{\tilde{P}^{(g)}_k(\textbf{v})\}_{k=1}^{5}$ was obtained by voxel-wise averaging of the registered labels: $\tilde{P}^{(g)}_k(\textbf{v}) = \frac{1}{N_g} \sum _{i=1}^{N_g} \mathbb {I}\!\left( Y_i(\textbf{v}) = k\right)$, where $N_g$ denotes the number of volumes in group *g* and $\mathbb {I}(\cdot )$ is the indicator function. To improve spatial coherence and mitigate local inconsistencies caused by registration errors or annotation noise, each preliminary probabilistic map was smoothed using a three-dimensional Gaussian kernel $\mathscr {G}_\sigma$: $P^{(g)}_k(\textbf{v}) = \left( \tilde{P}^{(g)}_k * \mathscr {G}_\sigma \right) (\textbf{v})$, where $\sigma =1.0$ controls the degree of smoothing. The resulting maps were subsequently normalized to satisfy $\sum _{k=1}^{5} P^{(g)}_k(\textbf{v}) = 1, \quad \forall \textbf{v}$.

For a given input CT scan, the image is registered to all reference images within each group, ensuring that at least one anatomically similar template is available. The quality of each registration is assessed using a composite similarity function ^35^ that combines Mean Squared Error (MSE), Normalized Cross-Correlation (NCC), and Mutual Information (MI). These metrics capture complementary aspects of image similarity, including intensity correspondence and statistical dependence. The final similarity score is computed as $w_{MSE} \cdot \frac{1}{1 + \text {MSE}} + w_{NCC} \cdot \frac{\text {NCC} + 1}{2} + w_{MI} \cdot \text {MI}$, where $w_{MSE}$, $w_{NCC}$, and $w_{MI}$ are weighting factors, all set to 1 in our implementation. The reference image achieving the highest score is selected as the most suitable probabilistic template for the input scan.

To accommodate substantial anatomical variability across CT volumes, non-rigid volumetric registration was performed using the DIPY library ^36^. As an initialization step, approximate alignment was achieved via center-of-mass matching (*transform_centers_of_mass*), which provides a robust pre-registration even under intensity differences. Subsequently, Symmetric Diffeomorphic Registration (SyN) ^37^ was applied to estimate a smooth, invertible deformation field. Mutual information was used as the similarity metric, as it is well suited for images with heterogeneous intensities, including scans with extensive pulmonary lesions, collapse, or poorly visible fissures. A multi-resolution strategy with pyramid iterations of [10000, 1000, 100], Gaussian smoothing, and progressive downsampling was employed to achieve consistent anatomical alignment, although performance may degrade in cases of extreme anatomical distortion or severe imaging artifacts.

The probabilistic templates are integrated at two stages of the proposed pipeline: they are provided as auxiliary inputs to **Stage 2** (high-resolution network) and are further exploited during the **post-processing stage**, where the most appropriate template is selected on a per-case basis (Fig. 1-c).

### LobePrior method

3D segmentation of the pulmonary lobes presents significant advantages over 2D approaches, mainly by leveraging spatial continuity across axial slices, allowing a more accurate representation of interlobar surfaces. Unlike 2D methods, which analyze isolated slices and may fail to capture incomplete or absent fissures, 3D models can integrate volumetric information, resulting in greater robustness against anatomical or pathological alterations such as lung collapse or extensive consolidations ^39^. This approach improves structural consistency in the segmentation and supports a comprehensive anatomical analysis of the lungs, being particularly valuable in clinical applications that require precise regional measurements or surgical planning ^10^.

Moreover, the present method was inspired by approaches that incorporate neighboring anatomical information ^12,14,34^ and employ multitasking with multiple decoders ^17,40,41^, strategies that have been shown to improve CNN performance, particularly in airway tree segmentation and in leveraging its anatomical relationship for lobe localization ^1,10^. Moreover, airway optimization is included as one of the subtasks of the LobePrior method. RPLS-Net ^40^, for instance, integrates the segmentation of fissures and lung lobes, enabling a more consistent and accurate anatomical representation, even in regions with poorly visible fissures. MEDPSeg ^17^, on the other hand, adopts a hierarchical strategy to simultaneously segment airways, vessels, lung parenchyma, and lesions, demonstrating robust performance even in cases of severe pulmonary disease.

In pulmonary lobe segmentation, it is advantageous for the network to learn both global and local features ^18^. Local features enable precise identification of lobe and fissure boundaries and allow adaptation to anatomical variations, such as differences in fissure thickness. Global features are useful when a fissure is not fully visible, aiding the network in capturing the overall lung structure and inter-lobar relationships. In the proposed approach, feature extraction is performed in two stages: a global analysis at low resolution, followed by a localized refinement at high resolution, focusing on critical regions via image patches.

The proposed method, called LobePrior (Fig. 1-c)), consists of three stages, designed to exploit different levels of spatial and anatomical information, progressing from coarse localization to detailed, respiratory structure-aware segmentation supported by prior information. The pipeline consists of: (i) an initial global low-resolution processing stage that overcomes memory limitations associated with high-resolution CT images, allowing the network to learn global lung features by considering the lung as a whole; (ii) a second high-resolution processing stage, specific to each lobe, the entire lung, and the airways, which refines the segmentation obtained in the first stage and accurately identifies and localizes incomplete or missing fissures caused by lesions (learning local features); and (iii) an post-processing step, where final voxel labels are derived from both network outputs and prior information.

The current LobePrior method extends the version presented at CBEB 2024 ^42^, incorporating several improvements: an expanded dataset including severe cases, a lesion insertion technique, a change from focal loss to Dice loss, probabilistic models for automatic group selection, and the architecture was redesigned, replacing five independent AttUNets ^41^ without weight sharing, which proved inefficient, with a single network comprising seven decoders that share weights. These updates enhanced the method’s efficiency and robustness, making it competitive with existing approaches.

During training each image is associated with a corresponding probabilistic model, according to the group to which it belongs. During inference of unseen images, the process begins with pre-processing and registration of the CT image to all reference images associated with the probabilistic models. The most suitable model is selected based on a similarity function ^43^, using non-rigid registration through the DIPY library ^36^. The registered image, along with the selected probabilistic model, is then provided to the pipeline.

**Stage 1 - Initial low-resolution segmentation:** the CT volume is processed at low resolution ($128 \times 128 \times 128$), resulting in a coarse lobar segmentation. This strategy reduces memory demand and allows the model to capture the global anatomical organization of the lungs, including lung location, the relative arrangement of the lobes, and relevant anatomical landmarks such as interlobar fissures and airways. An AttUNet ^41^, composed of an encoder and a decoder, takes as input a single CT volume and produces initial masks corresponding to the pulmonary lobes, the whole lung, and the airways. During training, the *Dice Loss* function is used in conjunction with the *softmax* activation. The resulting segmentation is employed to constrain the processing of subsequent stages to the lung region, while also providing an initial estimate of the interlobar boundaries.
**Stage 2 - Refined high-resolution segmentation:** the segmentation is locally refined at high resolution, with the aim of correcting incomplete or missing fissures, improving lobar boundaries, and addressing potential errors propagated from the previous stage. To this end, a second AttUNet is employed, composed of a shared encoder and seven independent decoders. The model inputs include: (i) the probabilistic model, represented by five channels, (ii) the original CT volume, and (iii) the seven masks generated in Stage 1, resulting in a total of 13 input channels. The output consists of seven segmentation maps corresponding to the whole lung, the five pulmonary lobes, and the airways. Training is performed using the *Dice Loss* function, with a sigmoid activation applied individually to the output of each decoder. At this stage, refinement is carried out through patch-based processing with patches of size $128 \times 128 \times 128$, enabling greater focus on local anatomical details. The probabilistic model is incorporated as an auxiliary probability map, provided as an additional input channel rather than as a hard constraint. In this way, it acts as a source of global anatomical context and expected lobar location, which is combined with local image information to guide an anatomically consistent refinement of the final segmentation.
**Stage 3 - Post-processing with prior information:** the outputs of the seven decoders are concatenated to form a single structure representing the pulmonary lobes and airways. Since each decoder generates a separate mask using the sigmoid function, the same voxel may be marked by more than one structure if its activation exceeds 0.5 in multiple decoders. In such cases, to determine which label the voxel should receive, the activation values are compared, and the label corresponding to the highest value is assigned, that is, the one for which the network is most confident. As softmax is not used, which is more appropriate for mutually exclusive classes, and each decoder is responsible for a specific structure, only positive sigmoid activations are considered to indicate the presence of targets. Consequently, the whole lung segmentation acts as a background indicator, and activations outside this region are disregarded. Then, the largest connected component is extracted from the binarized output of each decoder, corresponding to the whole lung, the pulmonary lobes, and the airways. Voxels within the lung region that are not assigned to any lobe are considered segmentation gaps, usually caused by severe abnormalities that result in low-confidence activations. These regions are filled based on the lobe indicated by the probabilistic model. Since the entire automated segmentation process performed by the LobePrior method occurs in a registered and isometric space, the final segmentation is transformed back to the original image space using a rigid transformation, and the original voxel resolution is restored via interpolation.

The proposed segmentation network adopts an encoder-decoder architecture inspired by the 3D U-Net. The input to the network is defined as the concatenation of the CT image volume $\textbf{X} \in \mathbb {R}^{H \times W \times D}$ and the probabilistic lobe prior $\textbf{P}$, which is treated as an additional input channel. Specifically, the augmented input is given by $\textbf{X}' = \textrm{Concat}(\textbf{X}, \textbf{P})$ and is fed directly into the first convolutional layer of the encoder. This formulation allows the network to jointly learn appearance-driven and anatomy-aware representations from the earliest stages of feature extraction. The subsequent encoder-decoder pathway and skip connections propagate this combined information, enabling robust lobe boundary delineation, particularly in regions where fissures are incomplete, displaced, or poorly visible due to severe pathological alterations.

Its important to highlight that our pulmonary segmentation targets from novel data include many cases with severe internal abnormal abnormalities in both the whole lung and lung lobe manual annotations. This teaches LobePrior to correctly identify lung regions with severe abnormality, and allows for any area inside the lung that was not confidently classified as a lobe by the network to be filled by the probabilistic model reference. In this way, the LobePrior methods includes contributions from 3D convolutional neural networks, neighboring anatomy guidance, and leverage of prior information from probabilistic models indicating expected lobe localization. The final segmentation consists of seven classes: background (BG), left lower lobe (LLL), left upper lobe (LUL), right lower lobe (RLL), right middle lobe (RML), right upper lobe (RUL), and airway.

### AttUNet

The variant of 3D AttUNet network used in this work was initially proposed by Carmo et al. ^41^. It is a CNN architecture based on the 3D U-Net ^44^, incorporating spatial attention mechanisms. The specific spatial attention module used in AttUNet was proposed by by Gorriz et al. ^45^ to assess the severity of knee osteoarthritis. AttUnet incorporates spatial attention modules to selectively focus on regions of interest of feature maps, by highlighting areas more relevant to the analysis trough learned sigmoid heatmaps.

### Synthetic lesion insertion and data augmentation

**Fig. 2:**
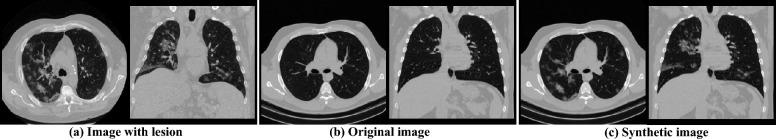
Example of lesion insertion in a training image: **(a)** coronal and transversal views of the original image containing the lesion (target); **(b)** selected representative original training image (source); and **(c)** resulting training image with the lesion inserted using various data augmentation techniques.

Lesion insertion is performed exclusively on the training phase data and is not applied during the testing phase (Fig. 2), in a type of data augmentation. The intent is to improve the quantity of representative cases of lobar segmentation including severe findings, contributing to data diversity ^16^. By exposing the network to a wider range of challenging scenarios, such as consolidations that obscure lobar fissures, synthetic lesions improve segmentation performance and generalization to real clinical cases. Lesions, computed by MEDPSeg ^17^, are extracted from a source image into a target image. The lesions are sourced from 20 pre-selected representative CT images of severe lung lesions. All lesion cases are registered to the probabilistic models. During training, one of the lesion CT images, registered to the appropriate template, is randomly selected and inserted into the target image. Since the images share the same space, the lesion can be incorporated within the pulmonary area of the target training image. Moreover, images with inserted lesions are used in both the first and second training stages. To smooth the transition between the base image and the insertion, a blending filter with soft edges was used. This involves applying a Gaussian blur to the edge of the mask in order to produce a more natural transition.

In addition, to ensure that inserted lesions have realistic intensity values, the lesion intensities are normalized to match the mean and standard deviation of the surrounding lung tissue in the target image before applying the Gaussian blending. This normalization preserves realistic HU ranges within the lesion and avoids introducing artificial intensity artifacts, while the Gaussian blending ensures smooth transitions at the lesion boundaries. Together, these steps maintain the global HU distribution and prevent bias toward specific lesion characteristics, ensuring that synthetic lesion insertion enhances data diversity without compromising realism or network generalization.

To further enhance the model’s variability, various commonly used data augmentation techniques are applied during network training, including random scaling and rotation, mirroring along all axes, Gaussian noise addition and blurring, brightness and contrast intensity adjustments, and finally, a random crop of $128 \times 128 \times 128$. Consequently, the lesion contained in the image is also affected by the transformations applied during data augmentation. All data augmentation techniques, including lesion insertion, are applied to the CT image, which serves as input to both stages of the method.

### Comparison with single-stage CNNs and Atlas-based methods

To contextualize LobePrior’s advantages, we contrast it with conventional single-stage CNNs and atlas-based approaches. Single-stage CNNs often struggle in regions with incomplete fissures or severe abnormalities, while atlas-based methods are highly sensitive to registration errors and anatomical variability.

LobePrior addresses these challenges by integrating probabilistic anatomical priors into a multi-stage CNN framework. This combination enables the model to leverage prior knowledge about lung anatomy while maintaining the flexibility of deep learning to adapt to image-specific features. As a result, LobePrior demonstrates superior robustness in handling cases with severe abnormalities, such as consolidations or nodules, and achieves higher segmentation accuracy compared to both single-stage CNNs and atlas-based pipelines. Furthermore, the multi-stage approach allows progressive refinement of predictions, correcting low-confidence regions in post-processing and ensuring consistent delineation of lung lobes across diverse datasets.

### Quantitative evaluation metrics

To evaluate the performance of the methods implemented on the test datasets, appropriate quantitative metrics were applied, calculated from the manual segmentations and volumetric predictions, both at the original image resolution, for each of the six labels (background and lung lobes). The following quantitative evaluation metrics are employed in this work: Dice Score ^46^, Average Hausdorff Distance (AHD) ^47^, and Absolute Volume Similarity (AVS) ^48^. These combined metrics provide a comprehensive assessment of segmentation quality, considering overlap, boundary precision, and volumetric difference, respectively ^48^.

Dice is used as an overlap metric between the predicted segmentation and the reference segmentation, taking into account false positives and false negatives. Note that Dice tends to assume a higher value in large structures, such as the pulmonary parenchyma, and the larger the structure, the less perceptible the penalty due to small errors ^48^. Average Hausdorff Distance measures the greatest distance between a point on the predicted segmentation boundary and the reference segmentation boundary. The Absolute Volume Similarity metric measures the absolute difference between the volumes of the predicted and reference segmentations, normalized by the reference volume (metric formulas are in the Supplementary Material).

### Training hyperparameters and software used

The choice of hyperparameters was guided by a set of preliminary experiments conducted with different configurations and a subset of the data, aiming to evaluate validation performance, ensure training stability, and prevent overfitting. As a result, the following settings were adopted: the AdamW optimizer ^49^ with an initial learning rate of $1e\text {-}4$, weight decay of $1e\text {-}5$, and exponential learning rate decay of 0.985; as well as the Dice loss function ^46^. The best weight, corresponding to the lowest validation loss on the 3D scans, is selected after approximately 50 training epochs on an NVIDIA GeForce RTX 4090 24 GB GPU.

All code was implemented in Python (3.10) using PyTorch Lightning (2.4.0) as the base deep learning framework. We also used several Python packages for data analysis and results visualization, including connected-components-3d (3.10.3), SimpleITK (2.4.0), nibabel (5.1.0), and torchio (0.20.1). More information is available in the GitHub repository: https://github.com/MICLab-Unicamp/LobePrior.

## Results

The airway was not evaluated, as the labels for this structure were generated during the training phase using the MEDPSeg method ^17^ to assist the network in lung lobe segmentation through neighboring anatomy guidance ^1^. Finally, qualitative assessments were conducted through the analysis of volumetric renderings of the worst cases, using the ground truth as a reference. In the experiments, all methods adopt 3D CNNs with the same training set and hyperparameters. To showcase the specific benefits of the proposed method to cases of severe abnormalities, we include results stratified by percentage of involvement, i.e, abnormality severity, as computed by MEDPSeg ^17^.

### Quantitative results

We conducted experiments on the LOCCA ^24^ dataset, which includes 60 CT images, as well as on the CT Images in COVID-19 and CoronaCases datasets, with 15 and 10 CT images, respectively (Table 2). For comparison, the LobePrior method was evaluated against publicly available approaches, namely nnU-Net ^20^, LungMask ^19^, and TotalSegmentor ^21^, all widely used for lung lobe segmentation and reflect the main methodological trends in the current state of the art. In addition to demonstrating competitive performance reported in the literature, these methods were selected based on their scientific relevance, widespread adoption in recent studies, and, fundamentally, their reproducibility. Whenever possible, we prioritized approaches with publicly available source code or sufficiently detailed methodological descriptions, enabling their implementation and fair comparison with the proposed method. This choice ensures that the experimental evaluation is transparent, rigorous, and aligned with best practices in deep learning–based research for medical image segmentation.

In particular, nnU-Net was retrained using the same training dataset employed for LobePrior, in order to ensure a fair comparison under identical data conditions. The remaining evaluated methods provide pretrained models together with their respective source code, enabling the direct prediction of new data. Consequently, these methods were applied to the same test sets and compared in a manner consistent with the proposed method.Only LobePrior employed probabilistic models and a network with multiple decoders, one for each lobe, the lungs, and the airways. LobePrior, TotalSegmentor, and LungMask refine their results using an auxiliary lung segmentation. LobePrior demonstrated notably robust performance in cases with severe pulmonary lesions, outperforming competing approaches. Detailed quantitative results for each lobe are presented in the Supplementary Material (Supplementary Table S3).

**Table 2:** Dice scores, Average Hausdorff Distance (mm), Absolute Volume Similarity, and Standard Deviation (STD) were evaluated on the test dataset, grouped by cancer and COVID-19 cases. Bold values indicate the best overall performance across all lobes, compared to other approaches.

Method	Dice±STD	AHD±STD	AVS±STD	Dice±STD	AHD±STD	AVS±STD
	LOCCA (Patients with COVID-19)			LOCCA (Patients with cancer)		
nnU-Net	0.889±0.054	2.836±2.510	0.930±0.054	0.953±0.022	0.483±0.451	0.977±0.018
LungMask	0.907±0.051	0.901±0.592	0.941±0.043	0.940±0.036	0.488±0.565	0.969±0.025
TotalSegmentor	0.939±0.033	0.301±0.265	0.965±0.029	0.956±0.025	0.189±0.181	0.977±0.019
LobePrior	**0.966**±**0.023**	**0.176**±**0.151**	**0.984**±**0.015**	**0.978**±**0.017**	**0.157**±**0.177**	**0.990**±**0.014**
	CT Images in COVID-19			CoronaCases		
nnU-Net	0.934±0.033	1.130±1.184	0.959±0.069	0.951±0.022	0.259±0.227	0.976±0.016
LungMask	0.950±0.028	0.270±0.198	0.976±0.023	0.953±0.025	0.263±0.205	0.973±0.020
TotalSegmentor	0.956±0.021	0.127±0.094	0.959±0.030	0.952±0.024	0.174±0.143	0.977±0.022
LobePrior	**0.987**±**0.008**	**0.039**±**0.046**	**0.997**±**0.003**	**0.978**±**0.014**	**0.086**±**0.074**	**0.992**±**0.007**

**Fig. 3:**
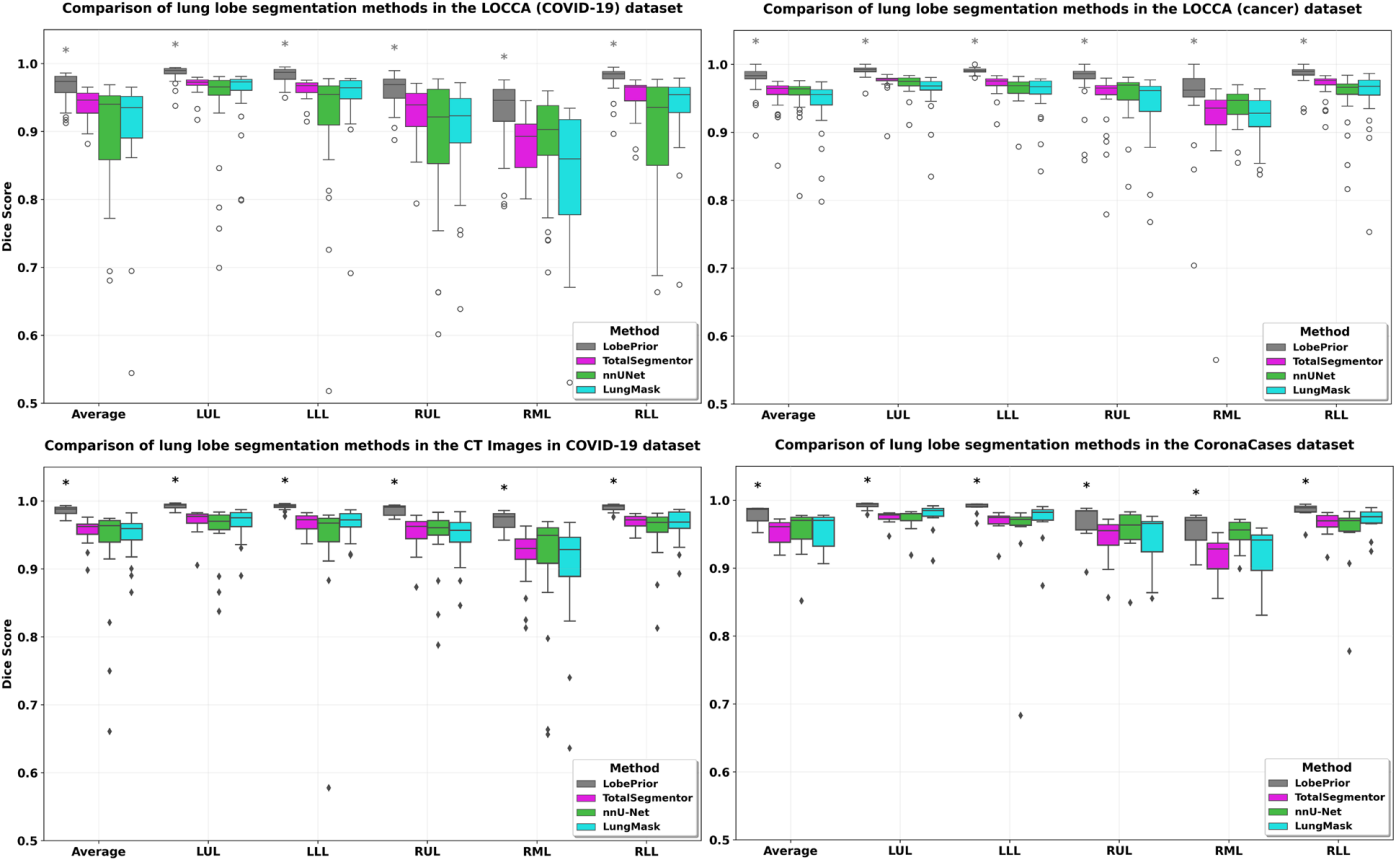
Dice scores per lobe for each test dataset. The LobePrior method demonstrated statistically significant superior performance (p < 0.05, Wilcoxon signed-rank test) compared with all other methods across all evaluated lobes. The asterisk (*) indicates statistical significance compared to the other methods.

Statistical comparisons using the Wilcoxon test with Holm–Bonferroni correction showed that LobePrior significantly outperformed the nnU-Net, LungMask, and TotalSegmentor methods in Dice score (adjusted p < 0.05 for all comparisons), particularly in the LUL, LLL, and RLL lobes, as well as in the overall mean Dice score across all five lobes (Fig. 3). In the CT Images in COVID-19 and CoronaCases datasets (Table 2 and Supplementary Table S5), which exhibit well-defined fissures compared to the other datasets, the LobePrior method achieved higher Dice scores for all lobes, standing out in the CoronaCases dataset, where it obtained a Dice score above 99% for all lobes.

Resulting Dice, AHD and AVS obtained in the LOCCA dataset, including their standard deviation, highlight that the proposed method achieved superior performance compared to competing approaches, particularly on the left side of the lung and in the RLL. All approaches exhibited outlier values in RML, as it is the most challenging lobe to segment. Higher standard deviation values were observed in the results obtained by the TotalSegmentor, LungMask, and nnU-Net methods.

In addition to the results reported in Table 2, Lessmann et al. ^2^ and Visvanathan et al. ^50^ evaluated their algorithms on 10 and 8 CT images, respectively, from the CoronaCases dataset ^15^. On the same dataset, the proposed LobePrior method achieved a mean Dice score of $0.978\pm 0.014$, which was significantly higher than that obtained by Lessmann et al. ^2^ ($0.947\pm 0.020$, p < 0.05) and statistically equivalent to the score reported by Visvanathan et al. ^50^ ($0.976\pm 0.015$, p > 0.05).

**Fig. 4:**
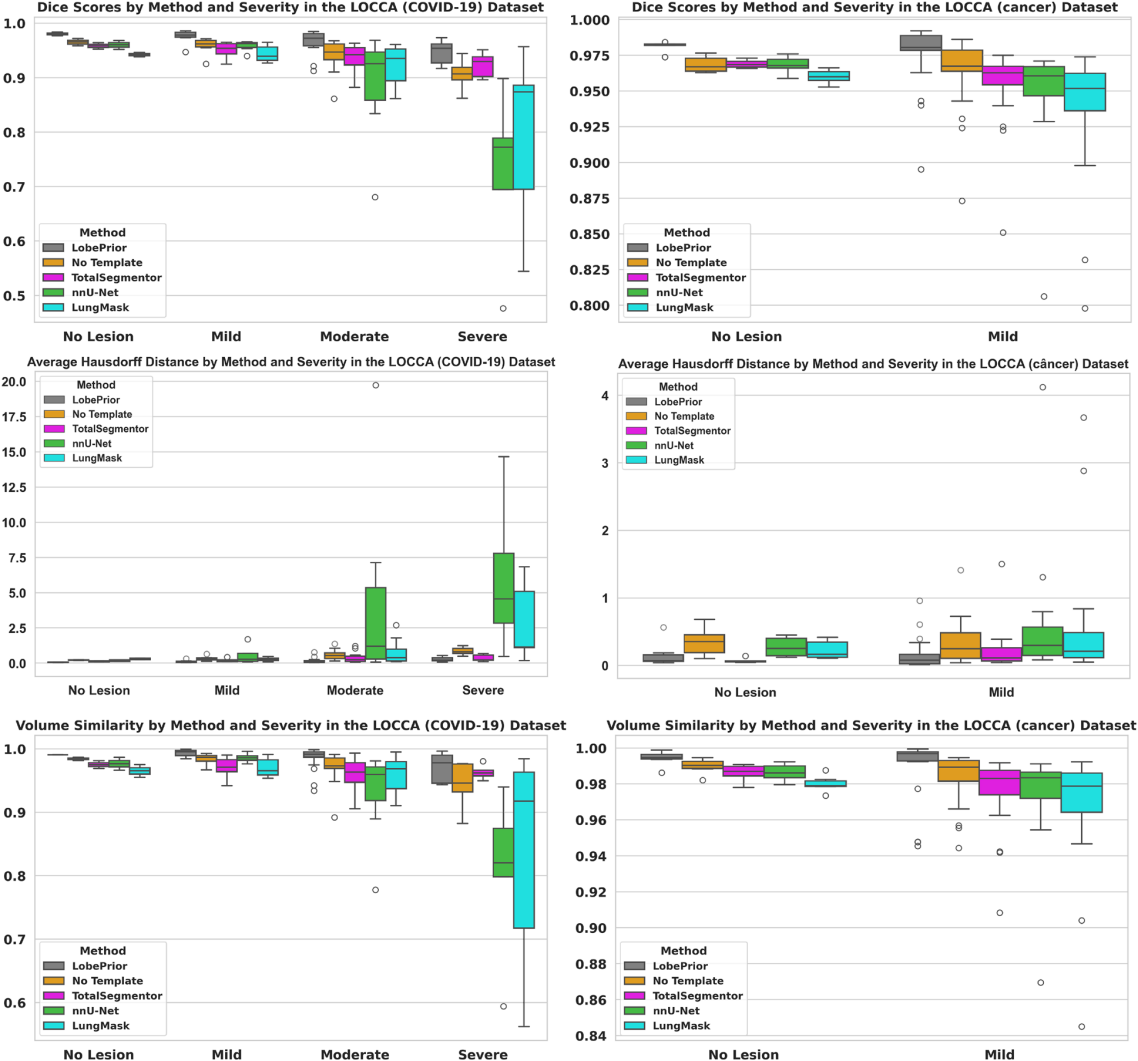
Boxplots of evaluations in the test dataset. On the left side of the figure, boxplots are presented with the Dice, AHD, and AVS scores for the LOCCA ^24^ dataset, categorized according to the degree of lung injury in COVID-19 patients. On the right side, the method is evaluated on the same dataset, but on images of cancer patients.

As expected, the severity of lung lesions significantly impacts segmentation accuracy, since severe alterations hinder the delineation of lobar fissures (Fig. 4). Despite a performance drop in cases with severe involvement, the proposed method, LobePrior, consistently outperforms competing approaches, demonstrating greater robustness under challenging conditions. The lower variability observed in the boxplots indicates higher stability and reliability of LobePrior. The combination of high volumetric scores (Dice and AVS) and low AHD values highlights the method’s effectiveness both in overall overlap and contour precision. Factors such as fibrosis and consolidations, common in severe lesions, increase this complexity, underscoring the importance of evaluating performance across different pulmonary impairment profiles.

The proposed method, named LobePrior, was initially evaluated using seven decoders, a configuration that achieved the best performance in segmenting both the airways and the whole lung, outperforming variations with fewer decoders. A version composed of five independent AttUnet networks without weight sharing was also tested, but the results were inferior. In addition, we assessed the effect of probabilistic post-processing, which significantly improved the segmentation accuracy. Finally, the inclusion of synthetic lesions exclusively during training contributed to enhancing the performance of the method in patients with diseased lungs. The complete results for these experiments are available in the Supplementary Material.

### Qualitative results

**Fig. 5:**
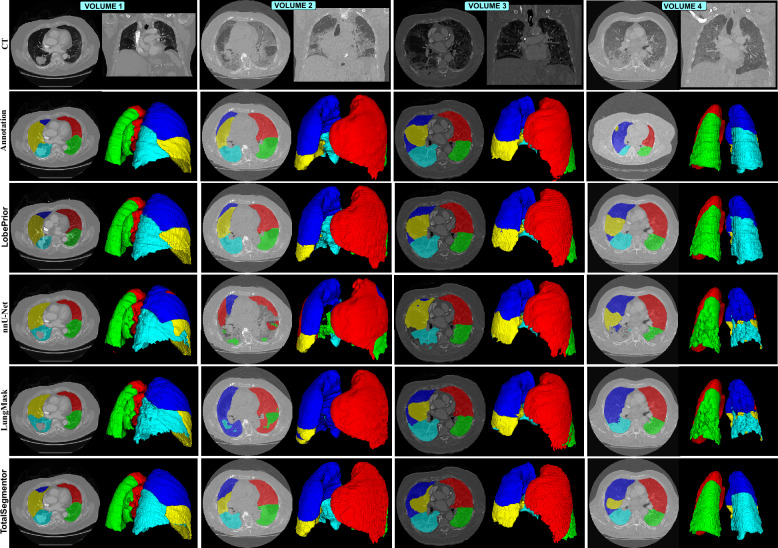
Qualitative results on CT images from the LOCCA dataset. Each column pair includes an axial view in the first column and a rendering of the lung lobes in the second, except for the first image in the second column, which is a CT image in the coronal view. The images in the second row correspond to manual annotations performed by the authors. The first two columns (volume 1) are from cancer patients, containing a moderate-type lesion. The remaining images are from COVID-19 patients and present severe-type lesions.

The qualitative results were included to visually illustrate the main advantages of the LobePrior method compared to existing approaches, particularly in cases with extensive pulmonary abnormalities. The images show that, even in the presence of incomplete or lesion-obstructed fissures, the probabilistic models enabled a more continuous and anatomically plausible reconstruction of the lung lobes, highlighting the LobePrior method’s ability to fill gaps based on prior anatomical knowledge. The 3D renderings (Fig. 5) display the predictions of the different networks alongside the manual annotations, allowing clear visual comparisons and highlighting cases in which conventional methods fail by interrupting segmentation in the absence of visible fissures, whereas LobePrior preserves the structural integrity of the lobes and is able to delineate the RML even in situations of low fissure visibility. In other cases (Supplementary Fig. S2 – qualitative evaluation on the NSCLC-Radiogenomics ^51^ dataset), it was observed that alternative approaches may adapt better to certain anatomical variations, leading to slightly more accurate segmentations. Notably, in Supplementary Fig. S2 (volumes 1 and 6), which present extensive lesions, LobePrior still produced consistent and anatomically coherent segmentations, reinforcing its robustness in challenging scenarios.

Segmentations generated by the LobePrior, TotalSegmentor, LungMask, and nnU-Net networks were compared in four cases with severe pulmonary impairment. The LobePrior network demonstrated superior performance in identifying lesioned regions, standing out from competing approaches. In particular, the use of a bounding box incorporated into the network input restricted the region of interest and minimized interference from adjacent tissues, facilitating the identification of lobar fissures and contributing to more accurate segmentation. Compared to the LobePrior version without probabilistic models, the method achieved statistically significant improvements in Dice score: up to 1.35% in cases with well-defined fissures and over 2.77% in cases with severe lung damage (Table 2). This improvement was especially evident when compared to TotalSegmentor, LungMask, and nnU-Net, which had difficulty accurately delineating anatomical structures. Relative to manual segmentation, LobePrior achieved results that were very close to the expected outcomes, standing out for its ability to preserve anatomical structures and reduce over- or under-segmentation errors caused by abnormalities.

### LOLA11 results

Although the LOLA11 challenge dataset contains images from non-COVID-19 patients, with less lung opacification. It is composed of CT scans with distorted lung areas, the presence of nodules, contrast agents, or noise, which makes accurate lung lobe segmentation challenging. So far, the LOLA11 lobe segmentation challenge has recorded 4 methods with mean overlap score in the range of [0.9280, 0.9226], 12 methods in the range of [0.9197, 0.9114], 6 methods in the range of [0.9088, 0.9017], and 11 methods in the range of [0.8993, 0.8900]. In total, more than 430 submissions were made to the LOLA11 challenge.

**Table 3:** Statistical summary of Dice similarity coefficients for each lung lobe on the LOLA11 test set. LL: left lung, RL: right lung, LLL: left lower lobe, LUL: left upper lobe, RLL: right lower lobe, RML: right middle lobe, RUL: right upper lobe. The overall mean Dice scores for the whole lung and for the lung lobes are reported at the bottom of the table.

Metric	LL	RL	LLL	LUL	RLL	RML	RUL
Q1	0.985	0.989	0.948	0.956	0.936	0.793	0.904
Q3	0.995	0.996	0.984	0.991	0.983	0.949	0.979
Max	0.997	0.998	0.994	0.997	0.995	0.997	0.999
Min	0.134	0.459	0.087	0.404	0.406	0.000	0.558
Std	0.118	0.088	0.221	0.125	0.107	0.254	0.107
Mean	0.965	0.968	0.901	0.938	0.930	0.799	0.915
Median	0.991	0.993	0.977	0.984	0.969	0.884	0.956

Regarding the LobePrior method (Table 3), the mean overlap score for the lungs was 0.9669 and for the pulmonary lobes was 0.8964, which is highly competitive when compared to state-of-the-art methods. The mean overlap score of LobePrior ranks among the top 30% of all submissions, being 0.0316 lower than that of the method by Bragman et al. ^52^ and 0.0291 lower than FissureNet ^18^, while outperforming the methods TotalSegmentator ^21^, LungMask ^19^, nnU-Net ^20^, and Zhang et al. ^8^.

### Computational performance

Benchmark tests conducted before image registration revealed an average GPU memory usage of 10.22 GB and a peak RAM usage of 4 GB, with an average execution time of approximately 34 minutes to process images with a large number of slices. This time corresponds to the first execution of registration, when the image alignment information is computed and stored locally. For subsequent predictions of the same image, this information is already available, drastically reducing the execution time to less than 1 minute and halving RAM usage. Similarly, for images with fewer slices, the first registration required approximately 14 minutes, with a GPU peak of 5 GB and RAM usage of 2 GB. When the prior registration is reused, the execution time drops to less than 22 seconds, while the GPU memory peak remains unchanged and RAM usage is halved. These results demonstrate that prior image registration significantly improves computational performance by leveraging stored information to accelerate subsequent predictions, regardless of image size.

### Ablation studies

To validate the contribution of each component of our framework, we conducted several ablation experiments, whose detailed results are presented in the Supplementary Material. Specifically, we evaluated the impact of (i) the application or not of post-processing (Supplementary Fig. S1 and Table S1), (ii) the inclusion or not of simulated lesions (Supplementary Table S2), (iii) the presence or absence of the probabilistic model (Fig. 4 and Supplementary Table S3), and (iv) different decoder configurations (Supplementary Table S4). Removing the probabilistic model resulted in a consistent decrease in Dice scores across all lobes, confirming its crucial role in guiding the segmentation, particularly in regions with incomplete or invisible fissures. The exclusion of simulated lesions slightly reduced robustness in severely damaged areas, highlighting their contribution to model generalization. In addition, the post-processing step improved the smoothness of lobe boundaries and reduced small segmentation artifacts. Moreover, employing multiple decoder paths enabled better feature fusion and produced more stable predictions compared with the single-decoder configuration. Together, these results reinforce the relevance of each component and the overall effectiveness of the proposed approach.

## Discussion

Probabilistic models paired with custom CNN architectures, along with data augmentation techniques and lesion insertion, enabled the development of a novel method CNN for high-quality pulmonary lobe segmentation in both healthy and abnormal cases. LobePrior achieves state-of-the-art performance in CT lung lobe image segmentation in cases of severe abnormality.

The quantitative results (Table 2 show that LobePrior achieved the highest Dice scores and low standard deviations, indicating more accurate segmentation and closer alignment with the reference segmentation. Additionally, it yielded lower AHD values, suggesting reduced discrepancies between the segmented and ground truth boundaries, and higher AVS scores, confirming greater fidelity to the true volume of the pulmonary structures. Altogether, these results, when compared with other approaches, indicate that the LobePrior method achieved superior performance in lung lobe segmentation. Despite not using probabilistic models, the approach employed in the development of the LobePrior method achieved performance superior to nnU-Net and LungMask, comparable to TotalSegmentor, but inferior to the version of LobePrior that incorporates probabilistic models.

Some of the external test sets, such as CoronaCases (n = 10), have relatively small sample sizes and exhibit substantial heterogeneity in terms of disease severity. These factors limit the statistical power of quantitative comparisons and restrict the extent of generalization. Rather than providing definitive evidence of clinical generalization, these experiments are intended to evaluate the robustness of the proposed method under particularly challenging and diverse conditions, including cases with severe abnormalities and poorly visible lobar fissures.

Methods such as LobePrior and LungMask rely on the lung mask during post-processing. If the lung mask is not accurately segmented, the lobe segmentation may lack precision. This could explain the inconsistent results obtained by the LungMask method compared to other approaches. In the LobePrior method, the probabilistic model-based hole filling contributed to more continuous segmentation in regions with abnormalities, as evidenced by improvements in quantitative overlap metrics and in qualitative analysis, such as in volume 1 (cancer) and volumes 2, 3, and 4 (COVID-19) shown in Fig. 5. This refinement was particularly advantageous in images of patients with severe lung lesions, where the presence of extensive opacities and anatomical distortions can hinder the accurate delineation of the lobes.

The segmentation of the right middle lobe remains the most challenging (as shown by the outliers in the RML lobe in Table 2 and Fig. 3). This difficulty can be attributed to the close proximity of the right middle lobe to the right upper and lower lobes. Moreover, the presence of incomplete lobar fissures further reduces segmentation accuracy. The right middle lobe is also small and subject to anatomical variations, making its segmentation particularly difficult. Across all datasets, especially in the LOCCA dataset involving COVID-19 patients, the difference in segmentation accuracy for the right middle lobe obtained by the method is relatively small, and the Dice standard deviation is reduced compared to other methods. These results indicate that the proposed LobePrior method is highly efficient and can effectively segment all lobes.

In the stratified analysis by injury severity (Fig. 4), the LobePrior method also stood out, delivering more consistent segmentations across different levels of pulmonary involvement, particularly in cases of severe lung injury. This validates a novel usage of registration and prior information in lung assessment as a way to fix segmentation problems caused by abnormalities, coupling a traditional registration based strategy, commonly used in older atlas based segmentation methods ^10^, with modern CNN based architectures. Additionally, benefits of using LobePrior on the accuracy of the segmentation in abnormal areas were also demonstrated in the qualitative assessment, highlighting LobePrior’s adaptability to the presence of opacities inside the lung.

Regardless of the test dataset (LOOCA, CT Images in COVID-19, and CoronaCases), the LobePrior method demonstrated superior performance for each lobe and in the overall average. In addition to outperforming all compared approaches across all metrics, it achieved statistically significant advantages over the TotalSegmentor, LungMask, and nnU-Net methods (Fig. 3).

LobePrior stands out by integrating probabilistic priors within a multi-stage CNN framework, enabling accurate lung lobe segmentation even in cases with incomplete fissures or severe pathologies. The inclusion of synthetic lesions during training further increases the diversity of scenarios presented to the model, overcoming common limitations of small or heterogeneous biomedical datasets. While networks such as UNet, UNETR, or Swin-UNet have achieved success in general segmentation tasks, their applicability is limited when anatomy is partially obscured. By combining deep learning with probabilistic guidance and synthetic data augmentation, LobePrior demonstrates greater robustness and generalization, distinguishing itself among recent biomedical image segmentation methods.

## Limitations

The LobePrior method can be trained on a GPU with 24 GB of memory and tested on a GPU with 12 GB of memory. The main limitation of this study is the high computational cost of image registration. Selecting the most suitable probabilistic model as prior information for a new test input is automated in the proposed pipeline. Registration errors may occur in highly deformed lungs, particularly in cases of severe pathological alterations or extensive tissue collapse. Such deformations can challenge non-rigid registration algorithms, leading to imperfect alignment between the priors and the target anatomy. However, in this failure mode, the resulting segmentation will not be significantly impacted, due to hole filling only being performed inside the lung area predicted by the network (Supplementary Table S1). The two-stage cascaded architecture, composed of convolutional blocks and specifically trained for pulmonary structure segmentation, together with the diversity of training data and the lesion insertion strategy, maintains high performance even in complex cases, ensuring accurate lobe segmentation. This effect is enhanced by the fact that the training includes cases with incomplete fissures, severe deformations, and significant anatomical variations, encouraging the network not to rely exclusively on probabilistic information for decision-making. As a result, the average improvements obtained by filling consolidated areas with the lobe label indicated by the prior information contribute to overall more consistent lobe segmentation (Fig. 5).

## Conclusion

This work proposes a method called LobePrior for the automatic segmentation of lung lobes in severely diseased lungs. This approach serves as an end-to-end lobe segmentation framework. We demonstrate that incorporating prior information can improve segmentation performance in cases presenting severe abnormalities, by on-the-spot correction of holes and under segmentation.

Experimental results have shown that the LobePrior method significantly improved lobe segmentation performance in CT images of patients with cancer and COVID-19. The average Hausdorff distance confirms that incorporating anatomical information is effective in achieving more accurate lobe boundaries. Higher Dice coefficients and Volume Similarity scores, compared to competing methods, indicate greater overlap between the predicted segmentation and the ground truth. This can also be observed in the qualitative results, where the lobe boundaries obtained by the proposed method are more consistent compared to the reference images. Other methods tend to fail when opacities are either on the lung border or cause incomplete fissures, an issue that is solved by our introduction of prior information from expected lung lobe position in a common space.

Overall, LobePrior proves to be a promising open-source option for analyzing computed tomography scans of patients with severe pulmonary impairment. The state-of-the-art performance of the method under these adverse conditions indicates its potential as a promising tool for clinical applications and research in advanced pulmonary diseases, although further evaluation in collaboration with medical professionals is needed to confirm its practical utility.

Future work will focus on reducing the computational complexity of the method and improving its generalization to different patterns of pulmonary abnormalities. In particular, the use of generative adversarial networks (GANs) will be investigated to directly learn and generate probabilistic lung lobe models, thereby eliminating the need for explicit image registration during the testing phase. This strategy has the potential to accelerate the inference pipeline while preserving anatomical consistency.

## Supplementary Information

Supplementary Information 1.

Supplementary Information 2.

Supplementary Information 3.

## Data Availability

The data are available within the manuscript, in the Materials and Methods section, or in the supplementary information files. The LobePrior method is publicly available on GitHub at https://github.com/MICLab-Unicamp/LobePrior, and it is also integrated into the MEDPSeg tool, available at https://github.com/MICLab-Unicamp/medpseg.

## References

[1] Doel, T., Gavaghan, D. J. & Grau, V. Review of automatic pulmonary lobe segmentation methods from CT. *Comput. Med. Imaging Graph.* **40**, 13–29. 10.1016/j.compmedimag.2014.10.008 (2015). 25467805 10.1016/j.compmedimag.2014.10.008

[2] Lessmann, N. et al. Automated Assessment of COVID-19 Reporting and Data System and Chest CT Severity Scores in Patients Suspected of Having COVID-19 Using Artificial Intelligence. *Radiology* **298**, E18–E28. 10.1148/radiol.2020202439 (2021). 32729810 10.1148/radiol.2020202439 PMC7393955

[3] Pang, H. et al. A fully automatic segmentation pipeline of pulmonary lobes before and after lobectomy from computed tomography images. *Comput. Biol. Med.* **147**, 105792. 10.1016/j.compbiomed.2022.105792 (2022). 35780601 10.1016/j.compbiomed.2022.105792

[4] Fedorov, A. et al. 3D slicer as an image computing platform for the quantitative imaging network. *Magn. Reson. Imaging* **30**, 1323–41. 10.1016/j.mri.2012.05.001 (2012). 22770690 10.1016/j.mri.2012.05.001 PMC3466397

[5] Yushkevich, P. A., Gao, Y., & Gerig, G. ITK-SNAP: An interactive tool for semi-automatic segmentation of multi-modality biomedical images. In,. *38th annual international conference of the IEEE engineering in medicine and biology society (EMBC), 3342–3345 (IEEE* 2016 (Orlando, USA, 2016). 10.1109/EMBC.2016.7591443 PMC5493443 28269019

[6] Lassen, B. et al. Automatic segmentation of the pulmonary lobes from chest CT scans based on fissures, vessels, and bronchi. *IEEE Trans. Med. Imaging* **32**, 210–222. 10.1109/TMI.2012.2219881 (2013). 23014712 10.1109/TMI.2012.2219881

[7] Wang, J., Betke, M. & Ko, J. P. Pulmonary fissure segmentation on CT. *Med. Image Anal.* **10**, 530–547. 10.1016/j.media.2006.05.003 (2006). 16807062 10.1016/j.media.2006.05.003 PMC2359730

[8] Zhang, Z. et al. Automatic segmentation of pulmonary lobes on low-dose computed tomography using deep learning. *Ann. Transl. Med.*10.21037/atm-20-5060 (2021). 33708918 10.21037/atm-20-5060 PMC7944332

[9] Yushkevich, P. et al. User-guided 3d active contour segmentation of anatomical structures: Significantly improved efficiency and reliability. *Neuroimage* **31**, 1116–1128 (2006). 16545965 10.1016/j.neuroimage.2006.01.015

[10] Carmo, D. S. et al. A systematic review of automated segmentation methods and public datasets for the lung and its lobes and findings on computed tomography images. *Yearb. Med. Inform.* **31**, 277–295. 10.1055/s-0042-1742517 (2022). 36463886 10.1055/s-0042-1742517 PMC9719778

[11] Xiao, H. et al. Deep learning-based lung image registration: A review. *Comput. Biol. Med.* **165**, 107434. 10.1016/j.compbiomed.2023.107434 (2023). 37696177 10.1016/j.compbiomed.2023.107434

[12] Van Rikxoort, E. M. et al. Automatic segmentation of the pulmonary lobes from fissures, airways, and lung borders: evaluation of robustness against missing data. In Yang, G., Hawkes, D., Rueckert, D., Noble, A. & Taylor, C. (eds.) *Medical Image Computing and Computer-Assisted Intervention – MICCAI 2009 : : 12th International Conference, London, UK, September 20-24, 2009, Proceedings, Part I*, vol. 1 of *Lecture Notes in Computer Science*, 263–271, 10.1007/978-3-642-04268-3_33 (Springer, Germany, 2009). 10.1007/978-3-642-04268-3_33 20425996

[13] Jurdi, R. E., Petitjean, C., Honeine, P. & Abdallah, F. BB-UNet: U-Net with bounding box prior. *IEEE J. Sel. Top. Signal Process.* **14**, 1189–1198. 10.1109/JSTSP.2020.3001502 (2020).

[14] Ma, J. et al. Segment anything in medical images. *Nat. Commun.* **15**, 654 (2024). 38253604 10.1038/s41467-024-44824-z PMC10803759

[15] Jun, M. et al. COVID-19 CT Lung and Infection Segmentation Dataset, 10.5281/zenodo.3757476 (2020).

[16] Pezeshk, A. et al. Seamless insertion of pulmonary nodules in chest CT images. *IEEE Transactions on Biomedical Engineering* **62**, 2812–2827. 10.1117/12.2043786 (2015). 26080378 10.1109/TBME.2015.2445054 PMC5547756

[17] Carmo, D. S. et al. MEDPSeg: Hierarchical polymorphic multitask learning for the segmentation of ground-glass opacities, consolidation, and pulmonary structures on computed tomography. arXiv preprint arXiv:2312.02365 (2024).

[18] Gerard, S. E., Patton, T., Christensen, G., Bayouth, J. & Reinhardt, J. FissureNet: A deep learning approach for pulmonary fissure detection in CT images. *IEEE Trans. Med. Imaging* **38**, 156–166. 10.1109/TMI.2018.2858202 (2019). 30106711 10.1109/TMI.2018.2858202 PMC6318012

[19] Hofmanninger, J. et al. Automatic lung segmentation in routine imaging is primarily a data diversity problem, not a methodology problem. *Eur. Radiol. Exp.* **4**, 50. 10.1186/s41747-020-00173-2 (2020). 32814998 10.1186/s41747-020-00173-2 PMC7438418

[20] Isensee, F., Jaeger, P. F., Kohl, S. A. A., Petersen, J. & Maier-Hein, K. H. nnUnet: A self-configuring method for deep learning-based biomedical image segmentation. *Nat. Methods* **18**, 203–211. 10.1038/s41592-020-01008-z (2021). 33288961 10.1038/s41592-020-01008-z

[21] Wasserthal, J. et al. TotalSegmentator: Robust segmentation of 104 anatomic structures in CT images. *Radiol. Artif. Intell.* **5**, e230024. 10.1148/ryai.230024 (2023). 37795137 10.1148/ryai.230024 PMC10546353

[22] Setio, A. A. A. et al. Validation, comparison, and combination of algorithms for automatic detection of pulmonary nodules in computed tomography images: The LUNA16 challenge. *Med. Image Anal.* **42**, 1–13 (2017). 28732268 10.1016/j.media.2017.06.015

[23] Tang, H., Zhang, C. & Xie, X. Automatic Pulmonary Lobe Segmentation Using Deep Learning. In *2019 IEEE 16th International Symposium on Biomedical Imaging (ISBI 2019)*, 1225–1228, 10.1109/ISBI.2019.8759468 (IEEE, Venice, Italy, 2019).

[24] Ribeiro, J. A. et al. Descriptor: Manually Annotated CT Dataset of Lung Lobes in COVID-19 and Cancer Patients (LOCCA). *IEEE Data Descriptions* **2**, 239–246. 10.1109/IEEEDATA.2025.3577999 (2025).

[25] An, P. et al. CT images in COVID-19 [data set]. *The Cancer Imaging Archive* **10**, 10.7937/TCIA.2020.GQRY-NC81 (2020).

[26] Zhang, M. et al. Multi-site, multi-domain airway tree modeling. *Med. Image Anal.*10.1016/j.media.2023.102957 (2023). 37716199 10.1016/j.media.2023.102957

[27] Afshar, P. et al. COVID-CT-MD, COVID-19 computed tomography scan dataset applicable in machine learning and deep learning. *Scientific Data* **8**, 121. 10.1038/s41597-021-00900-3 (2021). 33927208 10.1038/s41597-021-00900-3 PMC8085195

[28] Afshar, P. et al. Human-level COVID-19 diagnosis from low-dose CT scans using a two-stage time-distributed capsule network. *Sci. Rep.* **12**, 4827 (2022). 35318368 10.1038/s41598-022-08796-8 PMC8940967

[29] Grove, O. et al. Quantitative computed tomographic descriptors associate tumor shape complexity and intratumor heterogeneity with prognosis in lung adenocarcinoma. *PloS one* **10**, e0118261 (2015). 25739030 10.1371/journal.pone.0118261 PMC4349806

[30] Morozov, S. P. et al. Mosmeddata: Chest CT scans with COVID-19 related findings dataset. arXiv preprint arXiv:2005.06465 (2020).

[31] MedSeg, Jenssen, H. B. & Sakinis, T. MedSeg Covid Dataset 2, 10.6084/m9.figshare.13521509.v2 (2021).

[32] Zaffino, P. et al. An open-source COVID-19 CT dataset with automatic lung tissue classification for radiomics. *Bioengineering* **8**, 26 (2021). 33669235 10.3390/bioengineering8020026 PMC7919807

[33] Group, D. F. W. et al. Nifti:–neuroimaging informatics technology initiative (2004). https://nifti.nimh.nih.gov.

[34] Huang, H. et al. Medical Image Segmentation With Deep Atlas Prior. *IEEE Transactions on Medical Imaging* **40**, 3519–3530. 10.1109/TMI.2021.3089661 (2021). 34129495 10.1109/TMI.2021.3089661

[35] Zitová, B. & Flusser, J. Image registration methods: A survey. *Image Vis. Comput.* **21**, 977–1000. 10.1016/S0262-8856(03)00137-9 (2003).

[36] Contributors, D. et al. Dipy, a library for the analysis of diffusion MRI data. *Frontiers in Neuroinformatics* **8**, 8. 10.3389/fninf.2014.00008 (2014). 24600385 10.3389/fninf.2014.00008 PMC3931231

[37] Avants, B., Epstein, C., Grossman, M. & Gee, J. Symmetric diffeomorphic image registration with cross-correlation: Evaluating automated labeling of elderly and neurodegenerative brain. *Med. Image Anal.* **12**, 26–41. 10.1016/j.media.2007.06.004 (2008). 17659998 10.1016/j.media.2007.06.004 PMC2276735

[38] Betts, J. G. et al. *Anatomy and Physiology 2e* (OpenStax, Houston, Texas, 2022). Acesso gratuito - licença Creative Commons.

[39] Bao, N., Yuan, Y., Qingyao, L., Li, Q. & Zhang, L.-B. Edge-enhancement cascaded network for lung lobe segmentation based on ct images. *Front. Phys.* **11**, 1098756. 10.3389/fphy.2023.1098756 (2023).

[40] Liu, J. et al. RPLS-Net: Pulmonary lobe segmentation based on 3D fully convolutional networks and multi-task learning. *Int. J. Comput. Assist. Radiol. Surg.* **16**, 895–904. 10.1007/s11548-021-02360-x (2021). 33846890 10.1007/s11548-021-02360-x

[41] Carmo, D. S., Lotufo, R. A. & Rittner, L. MultiATTUNet: Brain Tumor Segmentation and Survival Multitasking. In *Brainlesion: Glioma, Multiple Sclerosis, Stroke and Traumatic Brain Injuries* (eds Crimi, A. & Bakas, S.) 424–434 (Springer International Publishing, 2021).

[42] Ribeiro, J. A., do Carmo, D. S., Reis, F. & Rittner, L. Deep Learning with Probabilistic Models for Segmenting Lung Lobes on Computed Tomography Images with Severe Abnormalities. In Soares, A. B., Leoni, R. F. & Cardoso, G. C. (eds.) *XXIX Brazilian Congress on Biomedical Engineering - Volume 3: Biomedical Informatics, and Biomedical Signal and Image Processing*, 243–252, 10.1007/978-3-031-94934-0_25 (Springer Nature Switzerland, Cham, 2025).

[43] Wang, Y., Yu, Q. & Yu, W. An improved Normalized Cross Correlation algorithm for SAR image registration. In *2012 IEEE International Geoscience and Remote Sensing Symposium*, 2086–2089, 10.1109/IGARSS.2012.6350961 (IEEE, Munich, Germany, 2012).

[44] Ö. Çiçek, Abdulkadir, A., Lienkamp, S. S., Brox, T. & Ronneberger, O. 3D U-Net: Learning Dense Volumetric Segmentation from Sparse Annotation. In Ourselin, S., Joskowicz, L., Sabuncu, M. R., Unal, G. & Wells, W. (eds.) *Medical Image Computing and Computer-Assisted Intervention – MICCAI 2016*, 424–432, 10.1007/978-3-319-46723-8_49 (Springer International Publishing, Cham, 2016).

[45] Górriz, M., Antony, J., McGuinness, K., Giró-i-Nieto, X. & O’Connor, N. E. Assessing Knee OA Severity with CNN attention-based end-to-end architectures. In Cardoso, M. J. et al. (eds.) *Proceedings of The 2nd International Conference on Medical Imaging with Deep Learning*, vol. 102 of *Proceedings of Machine Learning Research*, 197–214 (PMLR, 2019).

[46] Dice, L. R. Measures of the amount of ecologic association between species. *Ecology* **26**, 297–302. 10.2307/1932409 (1945).

[47] Aziz, A. A. T. & Hanbury, A. An efficient algorithm for calculating the exact Hausdorff distance. *IEEE Trans. Pattern Anal. Mach. Intell.* **37**, 2153–2163. 10.1109/TPAMI.2015.2408351 (2015). 26440258 10.1109/TPAMI.2015.2408351

[48] Taha, A. A. & Hanbury, A. Metrics for evaluating 3D medical image segmentation: Analysis, selection, and tool. *BMC Med. Imaging* **15**, 1–28. 10.1186/s12880-015-0068-x (2015). 26263899 10.1186/s12880-015-0068-x PMC4533825

[49] Loshchilov, I. & Hutter, F. Decoupled weight decay regularization. arXiv preprint arXiv:1711.05101 (2017).

[50] Visvanathan, M. et al. Assessing Lobe-wise Burden of COVID-19 Infection in Computed Tomography of Lungs using Knowledge Fusion from Multiple Datasets. In *2021 43rd Annual International Conference of the IEEE Engineering in Medicine & Biology Society (EMBC)*, 3961–3964, 10.1109/EMBC46164.2021.9629591 (2021). 10.1109/EMBC46164.2021.9629591 34892098

[51] Aerts, H. et al. Data From NSCLC-Radiomics [Data set]. The Cancer Imaging Archive. urlhttps://wiki.cancerimagingarchive.net/display/Public/NSCLC-Radiomics, 10.7937/K9/TCIA.2015.PF0M9REI (2019). **Acessado em 10/08/2022**.

[52] Bragman, F. J. S., McClelland, J. R., Jacob, J., Hurst, J. R. & Hawkes, D. J. Pulmonary lobe segmentation with probabilistic segmentation of the fissures and a groupwise fissure prior. *IEEE Trans. Med. Imaging* **36**, 1650–1663. 10.1109/TMI.2017.2688377 (2017). 28436850 10.1109/TMI.2017.2688377 PMC5547024

