# Supplementary material for: LobePrior segments lung lobes on computed tomography images in the presence of severe abnormalities
Source: Sci Rep. 2026 Apr 10;16:16205. doi: 10.1038/s41598-026-48136-8 (PMC13201581; doi:10.1038/s41598-026-48136-8)
Supplement: Supplementary file 1 — Supplementary Information 1. [file 41598_2026_48136_MOESM1_ESM.pdf]

Supplementary Material

for the manuscript entitled:
LobePrior Segments Lung Lobes on Computed Tomography Images in the Presence of Severe Abnormalities

Jean Antonio Ribeiro[1,*], Diedre Santos do Carmo[1], Fabiano Reis[2],
Ricardo Siufi Magalhães[3], Sergio San Juan Dertkigil[2], Simone Appenzeller[2],
Letícia Rittner[1]

[1]Universidade Estadual de Campinas, School of Electrical and Computer Engineering, Campinas, SP, 13083-970, Brazil
[2]Universidade Estadual de Campinas, School of Medical Sciences, Campinas, SP, 13083-970, Brazil
[3]Faculdade São Leopoldo Mandic, Department of Pulmonology, Campinas, SP, 13045-755, Brazil

[*]Corresponding author: Jean Antonio Ribeiro

[*] Corresponding author: j265739@dac.unicamp.br

Overview

This supplementary material contains additional details, figures, and tables that support the main manuscript.

Quantitative Evaluation Metrics

To quantitatively evaluate the performance of the proposed segmentation method, three widely adopted metrics were employed: *Dice Score*, *Average Hausdorff Distance (AHD)*, and *Absolute Volume Similarity (AVS)*. These metrics assess complementary aspects of segmentation accuracy, providing a comprehensive evaluation of overlap, boundary precision, and volumetric consistency. Together, these three metrics allow for a robust and multi-faceted evaluation of the segmentation performance, balancing overlap accuracy, surface delineation, and volumetric fidelity.

Dice Similarity Coefficient

The Dice Similarity Coefficient or Dice Score is a similarity measure that quantifies the overlap between the predicted segmentation S_p and the ground truth S_g. It is defined as:

$$DSC = \frac{2|S_p \cap S_g|}{|S_p| + |S_g|} \quad (1)$$

Dice values range from 0 to 1, where 1 indicates perfect agreement. This metric is particularly sensitive to the degree of overlap, making it widely used in medical image segmentation tasks.

Hausdorff Distance

The Hausdorff Distance (HD) evaluates the maximum boundary discrepancy between two sets of points, measuring how far the segmented surface is from the reference. The Average Hausdorff Distance (AHD) computes the average of the shortest distances from all points in one set to the other, making it less sensitive to outliers. For two point sets A (prediction) and B (ground truth), the Hausdorff Distance (HD) and is given by:

$$HD(A,B) = \max\left\{\sup_{a\in A}\inf_{b\in B}\|a-b\|, \sup_{b\in B}\inf_{a\in A}\|a-b\|\right\} \quad (2)$$

$$AHD(A,B) = \frac{1}{2}\left(\frac{1}{|A|}\sum_{a\in A}\min_{b\in B}|a-b|;+;\frac{1}{|B|}\sum_{b\in B}\min_{a\in A}|a-b|\right) \quad (3)$$

Lower HD and AHD values indicate better alignment of boundaries and fewer extreme segmentation errors. These metrics are particularly important in clinical applications where boundary precision is critical, such as the assessment of lobar fissures.

Absolute Volume Similarity

Absolute Volume Similarity (AVS) measures the consistency of segmented volumes, independently of spatial overlap, and is defined as:

$$AVS = 1 - \frac{|V_p - V_g|}{V_p + V_g} \tag{4}$$

where V_p and V_g represent the predicted and ground truth volumes, respectively. The AVS ranges from 0 to 1, with values closer to 1 indicating better volumetric agreement. This metric is particularly important for evaluating the preservation of anatomical structures in pathological cases.

Impact of Post-processing on Lung Lobe Segmentation

This section presents the results obtained before and after applying the post-processing step (Fig. S1 and Table S1). The procedure begins by applying the lung mask to exclude incorrectly classified voxels located outside the lung, followed by the selection of the largest connected component from the segmentation generated in the second stage of the method. Subsequently, any gaps are filled using voxels from the probabilistic model, where all voxels with a probability greater than 0.5 in each channel are assigned to the corresponding lobe. Finally, voxels outside the lung region are removed using the lung mask generated by one of the decoders of the proposed method. The results demonstrate that post-processing consistently improved the Dice scores across all lobes, highlighting its relevance in achieving more accurate segmentations. Table S1 also shows the results of applying the probabilistic model without including the probabilistic models, that is, by applying the lung mask to exclude incorrectly classified voxels located outside the lung, followed by the largest connected component selection.

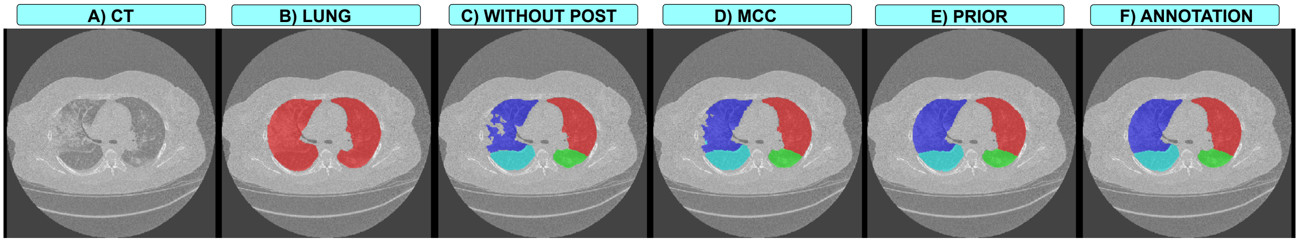

Supplementary Figure S1. Demonstration of post-processing applied to the network output: (A) CT image; (B) lung segmentation; (C) output of the LobePrior method without post-processing; (D) output after selecting the largest connected component (MCC) for each label; (E) result after post-processing with probabilistic models (template); (F) manual annotation of the lung lobes.

Effect of Synthetic Lesions on Lobe Segmentation Accuracy

The insertion of synthetic lesions during training led to a consistent improvement in lung lobe segmentation performance in patients with severe pulmonary injuries, as shown in Table S2. Compared to the network trained only with real images, this strategy enabled the model to learn more robust patterns in the presence of anatomical variations and regions with tissue destruction. This resulted in higher average Dice scores across all lobes when compared to the approach without lesion insertion. These findings indicate that synthetic lesion insertion enhances the model's generalization ability in adverse clinical scenarios by simulating situations that are underrepresented in the original dataset.

Quantitative Results on LOCCA, CT Images COVID-19, and CoronaCases Datasets

To evaluate the effectiveness of the proposed method, we examined its performance on three datasets exclusively selected for testing: LOCCA (including COVID-19 and lung cancer cases), CT Images COVID-19, and CoronaCases. Quantitative results for each pulmonary lobe individually, as well as the overall mean, are presented in Table S3. The metrics include the Dice Similarity Coefficient (DSC), Hausdorff Distance (HD), and Average Surface Distance (AVS), providing complementary perspectives on volumetric overlap and boundary accuracy.

These results allow a detailed analysis of the method's ability to delineate each lobe under challenging conditions, such as severe parenchymal abnormalities, while also demonstrating its generalization capability across different datasets with varying pathologies and acquisition protocols.

Average±STD	LUL	LLL	RUL	RML	RLL	Post-processing
0.919±0.027	0.952	0.911	0.898	0.901	0.868	no
0.913±0.053	0.959	0.923	0.910	0.831	0.941	without probabilistic model
0.927±0.044	0.960	0.958	0.905	0.849	0.964	with a probabilistic model
0.937±0.025	0.972	0.940	0.953	0.923	0.899	no
0.956±0.031	0.981	0.967	0.960	0.907	0.964	without probabilistic model
0.962±0.025	0.985	0.975	0.964	0.914	0.971	with a probabilistic model
0.879±0.069	0.910	0.902	0.886	0.746	0.948	no
0.963±0.015	0.975	0.971	0.948	0.950	0.971	without probabilistic model
0.974±0.010	0.985	0.976	0.972	0.956	0.978	with a probabilistic model
0.858±0.057	0.933	0.806	0.808	0.821	0.923	no
0.914±0.054	0.931	0.944	0.918	0.810	0.969	without probabilistic model
0.917±0.066	0.938	0.950	0.927	0.790	0.980	with a probabilistic model
0.898±0.056	0.971	0.866	0.945	0.897	0.812	no
0.945±0.021	0.950	0.959	0.943	0.956	0.919	without probabilistic model
0.954±0.017	0.977	0.965	0.954	0.947	0.926	with a probabilistic model

Supplementary Table S1. Dice scores for CT images at two time points: before and after post-processing. In this table, five computed tomography (CT) volumes were randomly selected.

Average±STD	LUL	LLL	RUL	RML	RLL	Synthetic Images
0.943±0.041	0.968	0.975	0.941	0.865	0.968	no
0.917±0.066	0.938	0.950	0.927	0.790	0.980	yes
0.912±0.075	0.970	0.969	0.894	0.773	0.952	no
0.927±0.044	0.960	0.958	0.905	0.849	0.964	yes
0.967±0.017	0.986	0.979	0.964	0.938	0.969	no
0.974±0.010	0.985	0.976	0.972	0.956	0.978	yes
0.945±0.025	0.975	0.962	0.941	0.901	0.947	no
0.962±0.025	0.985	0.975	0.964	0.914	0.971	yes
0.942±0.033	0.980	0.970	0.942	0.933	0.886	no
0.954±0.017	0.977	0.965	0.954	0.947	0.926	yes

Supplementary Table S2. Dice scores for different CT images from the LOCCA dataset, corresponding to patients with COVID-19 and severe lung injuries, comparing the results of the network trained with and without the insertion of synthetic lesions. In this table, five computed tomography (CT) volumes were randomly selected.

Performance of the different decoder variations in the LobePrior method

The performance of the method using 5 AttU-Nets, each with its own encoder and decoder, was inferior to that of methods with one or more shared decoders. In the method with 5 AttU-Nets, the weights of each network are not shared, whereas in the decoder-based methods, the weights are shared during the encoding stage. Networks with 6 or 7 encoders benefit from segmenting the lungs and airways, which can aid in lobe segmentation, resulting in improved performance for the network called LobePrior (Table S4).

Method	Lobe	LOCCA COVID			LOCCA Cancer			CT Images COVID			CoronaCases		
		Dice±STD	AHD±STD	AVS±STD	Dice±STD	AHD±STD	AVS±STD	Dice±STD	AHD±STD	AVS±STD	Dice±STD	AHD±STD	AVS±STD
nnU-Net	LUL	0.940±0.070	1.900±3.941	0.975±0.039	0.971±0.014	0.255±0.359	0.991±0.010	0.958±0.037	0.827±2.531	0.982±0.033	0.971±0.020	0.120±0.021	0.989±0.152
	LLL	0.916±0.095	2.654±8.271	0.947±0.062	0.963±0.019	0.578±1.241	0.979±0.019	0.942±0.080	2.385±9.459	0.958±0.071	0.940±0.091	0.261±0.038	0.970±0.603
	RUL	0.885±0.102	5.329±9.777	0.946±0.078	0.954±0.034	0.393±0.435	0.978±0.033	0.948±0.047	0.661±1.266	0.971±0.049	0.952±0.040	0.441±0.026	0.980±1.011
	RML	0.856±0.132	1.816±2.790	0.896±0.133	0.924±0.090	0.715±2.024	0.961±0.053	0.891±0.141	0.985±1.947	0.934±0.131	0.949±0.024	0.190±0.014	0.979±0.161
	RLL	0.851±0.212	2.480±4.622	0.886±0.202	0.953±0.038	0.474±0.603	0.977±0.033	0.930±0.137	0.790±2.149	0.950±0.135	0.944±0.063	0.284±0.063	0.962±0.448
	Average	0.889±0.054	2.836±2.510	0.930±0.054	0.953±0.022	0.483±0.451	0.977±0.018	0.934±0.033	1.130±1.184	0.959±0.069	0.951±0.022	0.259±0.227	0.976±0.016
LungMask	LUL	0.955±0.045	0.438±1.230	0.976±0.040	0.960±0.029	0.147±0.201	0.981±0.030	0.968±0.022	0.179±0.291	0.984±0.019	0.975±0.025	0.090±0.088	0.987±0.088
	LLL	0.935±0.099	0.657±1.804	0.959±0.100	0.957±0.030	0.195±0.388	0.977±0.016	0.967±0.019	0.285±0.742	0.987±0.014	0.968±0.035	0.214±0.029	0.982±0.408
	RUL	0.896±0.075	1.399±2.382	0.946±0.069	0.941±0.049	0.478±0.937	0.965±0.048	0.948±0.032	0.275±0.427	0.976±0.032	0.939±0.046	0.387±0.044	0.962±0.514
	RML	0.836±0.098	1.137±1.087	0.880±0.108	0.889±0.140	1.326±3.160	0.947±0.103	0.900±0.077	0.452±0.490	0.948±0.069	0.916±0.048	0.403±0.044	0.949±0.432
	RLL	0.911±0.152	0.875±1.927	0.943±0.154	0.954±0.044	0.294±0.600	0.977±0.017	0.966±0.023	0.159±0.227	0.984±0.013	0.969±0.021	0.219±0.020	0.984±0.209
	Average	0.907±0.051	0.901±0.592	0.941±0.043	0.940±0.036	0.488±0.565	0.969±0.025	0.950±0.028	0.270±0.198	0.976±0.023	0.953±0.025	0.263±0.205	0.973±0.020
TotalSegmentor	LUL	0.933±0.072	1.219±2.893	0.964±0.046	0.962±0.024	0.246±0.281	0.981±0.021	0.961±0.025	0.217±0.348	0.982±0.021	0.971±0.023	0.129±0.027	0.987±0.103
	LLL	0.906±0.097	2.002±5.212	0.944±0.080	0.958±0.033	0.429±0.713	0.978±0.024	0.953±0.042	0.371±0.857	0.979±0.025	0.959±0.040	0.250±0.039	0.981±0.278
	RUL	0.870±0.098	3.264±6.759	0.936±0.085	0.951±0.036	0.359±0.621	0.975±0.034	0.942±0.037	0.240±0.428	0.968±0.037	0.946±0.033	0.423±0.030	0.975±0.831
	RML	0.848±0.115	1.302±2.108	0.887±0.118	0.922±0.094	0.632±1.838	0.960±0.056	0.896±0.123	0.492±1.042	0.940±0.123	0.945±0.028	0.185±0.016	0.976±0.192
	RLL	0.853±0.192	1.980±3.544	0.890±0.185	0.950±0.039	0.438±0.546	0.977±0.032	0.934±0.123	0.206±0.265	0.957±0.113	0.948±0.061	0.276±0.056	0.971±0.388
	Average	0.882±0.055	1.953±2.041	0.924±0.047	0.948±0.028	0.421±0.493	0.974±0.024	0.937±0.024	0.305±0.256	0.965±0.021	0.954±0.022	0.253±0.091	0.978±0.018
Approach without Probabilistic Models	LUL	0.918±0.078	1.546±3.214	0.956±0.050	0.957±0.027	0.282±0.387	0.978±0.027	0.952±0.033	0.351±0.661	0.975±0.033	0.963±0.029	0.144±0.026	0.982±0.149
	LLL	0.892±0.108	2.234±5.893	0.936±0.087	0.950±0.034	0.467±0.863	0.973±0.029	0.944±0.043	0.392±0.835	0.970±0.043	0.952±0.041	0.241±0.045	0.975±0.301
	RUL	0.865±0.100	3.392±6.965	0.929±0.087	0.946±0.037	0.373±0.600	0.972±0.035	0.939±0.037	0.267±0.456	0.966±0.037	0.943±0.035	0.437±0.033	0.972±0.861
	RML	0.841±0.123	1.418±2.319	0.881±0.124	0.918±0.093	0.671±1.913	0.959±0.057	0.893±0.123	0.493±1.042	0.939±0.123	0.941±0.031	0.191±0.015	0.974±0.198
	RLL	0.851±0.189	1.980±3.544	0.890±0.185	0.950±0.039	0.438±0.546	0.977±0.032	0.934±0.123	0.206±0.265	0.957±0.113	0.948±0.061	0.276±0.056	0.971±0.388
	Average	0.873±0.052	2.114±2.110	0.918±0.045	0.944±0.027	0.446±0.446	0.972±0.024	0.932±0.029	0.342±0.216	0.961±0.024	0.949±0.024	0.258±0.094	0.975±0.019
LobePrior	LUL	**0.975±0.026**	**0.270±0.650**	**0.988±0.019**	**0.981±0.015**	**0.110±0.123**	**0.993±0.009**	**0.979±0.014**	**0.078±0.101**	**0.990±0.010**	**0.983±0.013**	**0.045±0.010**	**0.992±0.007**
	LLL	**0.961±0.041**	**0.410±0.958**	**0.981±0.033**	**0.975±0.022**	**0.196±0.283**	**0.987±0.015**	**0.974±0.018**	**0.094±0.142**	**0.989±0.014**	**0.976±0.020**	**0.079±0.018**	**0.989±0.009**
	RUL	**0.960±0.030**	**0.450±0.981**	**0.986±0.024**	**0.972±0.023**	**0.162±0.222**	**0.989±0.012**	**0.973±0.012**	**0.073±0.102**	**0.990±0.008**	**0.975±0.016**	**0.062±0.014**	**0.991±0.009**
	RML	**0.930±0.041**	**0.303±0.562**	**0.966±0.038**	**0.958±0.032**	**0.288±0.564**	**0.976±0.025**	**0.958±0.018**	**0.073±0.105**	**0.980±0.017**	**0.964±0.022**	**0.046±0.009**	**0.986±0.012**
	RLL	**0.950±0.038**	**0.308±0.631**	**0.972±0.034**	**0.972±0.017**	**0.123±0.145**	**0.989±0.009**	**0.973±0.017**	**0.072±0.103**	**0.989±0.012**	**0.977±0.017**	**0.049±0.012**	**0.991±0.007**
	Average	**0.955±0.021**	**0.348±0.230**	**0.978±0.014**	**0.972±0.018**	**0.176±0.074**	**0.987±0.008**	**0.971±0.014**	**0.078±0.014**	**0.988±0.009**	**0.975±0.016**	**0.056±0.013**	**0.990±0.007**

Supplementary Table S3. Dice scores, Average Hausdorff Distance (AHD) (mm), Absolute Volume Similarity (AVS), and Standard Deviation (STD) values for the LOCCA (COVID-19 and Cancer), CT Images COVID-19, and CoronaCases datasets. Bold values indicate the best performance for each lung lobe and the overall average.

Method	Average	LUL	LLL	RUL	RML	RLL
			**Dice Score±STD**			
1 decoder	0.961±0.021	0.984±0.011	0.981±0.011	0.952±0.030	0.912±0.051	0.975±0.021
2 decoders	0.963±0.022	0.985±0.010	0.982±0.012	0.955±0.035	0.922±0.045	0.974±0.024
5 U-Nets	0.947±0.041	0.981±0.009	0.977±0.014	0.939±0.037	0.874±0.056	0.963±0.029
5 decoders	0.954±0.024	0.976±0.025	0.969±0.035	0.946±0.043	0.916±0.061	0.962±0.033
6 decoders	0.957±0.024	0.981±0.012	0.977±0.017	0.947±0.036	0.908±0.057	0.971±0.024
7 decoders	**0.966±0.023**	**0.986±0.012**	**0.983±0.012**	**0.958±0.032**	**0.927±0.051**	**0.976±0.026**
			**Average Hausdorff Distance±STD**			
1 decoder	0.209±0.169	0.057±0.062	0.079±0.084	0.286±0.281	0.454±0.390	0.171±0.215
2 decoders	0.198±0.197	0.071±0.050	0.077±0.086	0.250±0.335	0.392±0.364	0.162±0.159
5 U-Nets	0.391±0.295	0.521±0.760	0.135±0.280	0.442±0.669	0.393±0.499	0.465±0.416
5 decoders	0.321±0.298	0.068±0.063	0.105±0.177	0.372±0.366	0.817±0.855	0.244±0.351
6 decoders	0.224±0.215	0.070±0.115	0.093±0.130	0.283±0.272	0.498±0.497	0.175±0.324
7 decoders	**0.176±0.151**	**0.056±0.084**	**0.072±0.084**	**0.199±0.215**	**0.393±0.399**	**0.159±0.323**
			**Absolute Volume Similarity±STD**			
1 decoder	0.980±0.018	0.994±0.011	0.993±0.010	0.973±0.029	0.951±0.050	0.990±0.012
2 decoders	0.981±0.019	0.993±0.018	0.992±0.019	0.975±0.036	0.961±0.043	0.985±0.018
5 U-Nets	0.978±0.016	0.985±0.025	0.981±0.032	0.980±0.023	0.961±0.056	0.980±0.023
5 decoders	0.975±0.022	0.990±0.011	0.989±0.013	0.971±0.031	0.938±0.065	0.986±0.017
6 decoders	0.979±0.020	0.994±0.009	0.992±0.011	0.973±0.032	0.952±0.054	0.985±0.022
7 decoders	**0.984±0.015**	**0.993±0.013**	**0.992±0.013**	**0.979±0.028**	**0.963±0.052**	**0.990±0.010**

Supplementary Table S4. Dice scores and standard deviations (STD) obtained for COVID-19 patients in the LOCCA test set, considering different methods. Bold values indicate the best performance for each lung lobe and for the overall average, compared with other approaches.

Qualitative Evaluation of Segmentation in the NSCLC Dataset

In Figure S2, particularly in volumes 3 and 6, this scenario can be observed: in volume 6, the LobePrior method successfully segmented the right middle lobe (RML), even under challenging conditions. In contrast, the regions corresponding to the upper lobes tend to be segmented more easily by all methods, as the fissures are more visible and present higher anatomical contrast. LobePrior demonstrated advantages in situations where fissures were partially invisible or interrupted, benefiting from the probabilistic model that guides the network in these uncertain regions. Other methods showed competitive performance in cases with more visible fissures but tend to fail when anatomical boundaries are not well defined. Notably, in Supplementary Fig. S2 (volumes 1 and 6), which present extensive lesions, LobePrior still produced consistent and anatomically coherent segmentations, reinforcing its robustness in challenging scenarios.

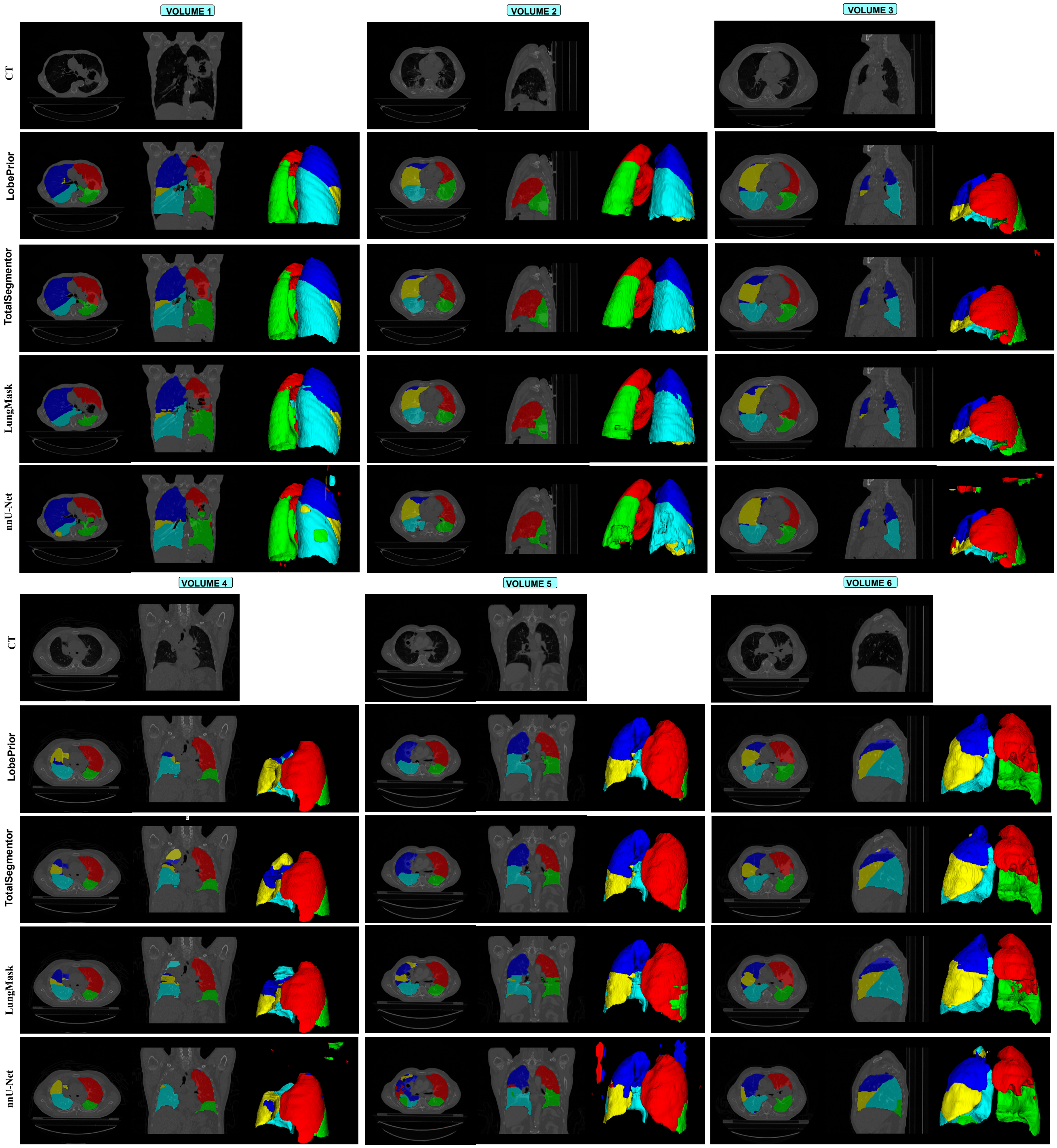

Supplementary Figure S2. Qualitative comparison of lung lobe segmentation results on NSCLC cases.

Generation of Probabilistic Anatomical Models

The generation of probabilistic anatomical models aims to capture the structural variability of the lung lobes across different subjects and acquisition conditions. To achieve this, a set of CT volumes and their corresponding lobe annotations are spatially aligned through non-rigid registration, enabling voxel-wise correspondence across all cases. The aligned images are then grouped based on structural similarity to ensure that each group represents a distinct anatomical configuration. Within each group, probabilistic maps are computed by estimating, for every voxel, the likelihood of belonging to each lobe. The resulting set of models $P_1, P_2, \ldots, P_n$ thus encodes the distribution of anatomical variability present in the dataset. The complete procedure is summarized in Algorithm 1, which details the algorithmic flow for the generation of probabilistic models.

Supplementary Algorithm 1: Generation of Probabilistic Models from Volumetric CT Images

---

Input: Set of pre-processed volumetric CT images $\{I_1, I_2, \ldots, I_{50}\}$
Corresponding lung lobe masks $\{L_1, L_2, \ldots, L_{50}\}$
Output: Set of probabilistic models $P_1, P_2, \ldots, P_n$ representing different anatomical configurations of the lung lobes, in which each model defines the probability of each voxel belonging to one of the lobes

Step 1: Non-rigid registration
foreach *pair* (I_i, I_j) *in* $\{I\}$ **do**
    Register $I_i \rightarrow I_j$ using a non-rigid transformation T_{ij};
    Apply T_{ij} to L_i to obtain aligned masks;

Step 2: Dice similarity computation
foreach *pair of registered masks* (L_i, L_j) **do**
    Compute $\text{Dice}(L_i, L_j)$;

Step 3: Structural grouping based on similarity
Initialize an empty list of groups $\mathcal{G} \leftarrow \emptyset$;
foreach *pair* (L_i, L_j) **do**
    if $\text{Dice}(L_i, L_j) > 0.8$ **then**
        if L_i *or* L_j *belong to an existing group* $g_k \in \mathcal{G}$ **then**
            Assign both L_i and L_j to that group g_k;
        else
            Create a new group g_{new} containing (L_i, L_j);
            Add g_{new} to $\mathcal{G}$;

Step 4: Generation of probabilistic models
foreach *group* $g_k \in \mathcal{G}$ **do**
    Initialize $P_k = 0$;
    foreach *voxel* v **do**
        $P_k(v) =$ mean of voxel intensities across registered masks in g_k;
    Add P_k to $\{P\}$;

Inference phase

Model selection for inference
for *a new input image* I_{test} **do**
    Register I_{test} with all probabilistic models P_k;
    Select the model P_k with the highest similarity score (e.g., Dice);
    Use the selected model to guide the segmentation of I_{test};

Table S5 presents the Dice scores, standard deviation (STD), and p-values for each lung lobe across the four evaluated datasets: LOCCA COVID, LOCCA Cancer, CT Images COVID, and CoronaCases. It can be observed that the LobePrior method consistently achieved the highest Dice values for all lobes and datasets, with the average values per method statistically outperforming all competitors, as indicated by the individual p-values. These results demonstrate that the integration of cascade convolutional networks with probabilistic models and the lesion insertion strategy allows LobePrior to maintain superior performance even in challenging cases, including incomplete fissures, severe pulmonary deformations, and significant anatomical variations. The statistical significance further supports the robustness and reliability of the method compared to approaches based on nnU-Net, LungMask, TotalSegmentator, and methods without probabilistic models.

Method	Lobe	LOCCA COVID (n = 30 cases)		LOCCA Cancer (n = 30 cases)		CT Images COVID (n = 15 cases)		CoronaCases (n = 10 cases)	
		Dice±STD	p-value	Dice±STD	p-value	Dice±STD	p-value	Dice±STD	p-value
nnU-Net	LUL	0.940±0.070	0.0008	0.971±0.014	< 0.0001	0.958±0.037	< 0.0001	0.971±0.020	0.0249
	LLL	0.916±0.095	0.0003	0.963±0.019	< 0.0001	0.942±0.080	< 0.0001	0.940±0.091	0.0002
	RUL	0.885±0.102	0.0002	0.954±0.034	< 0.0001	0.948±0.047	0.0098	0.952±0.040	0.0015
	RML	0.856±0.132	0.0110	0.924±0.090	0.0038	0.891±0.141	0.0002	0.949±0.024	0.0032
	RLL	0.851±0.212	0.0018	0.953±0.038	< 0.0001	0.930±0.137	< 0.0001	0.944±0.063	0.0028
	Average	0.889±0.054	0.0004	0.953±0.022	< 0.0001	0.934±0.033	< 0.0001	0.951±0.022	0.0010
LungMask	LUL	0.955±0.045	0.0007	0.960±0.029	< 0.0001	0.968±0.022	< 0.0001	0.975±0.025	< 0.0001
	LLL	0.935±0.099	0.0114	0.957±0.030	< 0.0001	0.967±0.019	< 0.0001	0.968±0.035	< 0.0001
	RUL	0.896±0.075	< 0.0001	0.941±0.049	< 0.0001	0.948±0.032	< 0.0001	0.939±0.046	< 0.0001
	RML	0.836±0.098	< 0.0001	0.889±0.140	0.0010	0.900±0.077	0.0020	0.916±0.048	< 0.0001
	RLL	0.911±0.152	0.0202	0.954±0.044	0.0005	0.966±0.023	0.0010	0.969±0.021	< 0.0001
	Average	0.907±0.051	0.0004	0.940±0.036	< 0.0001	0.950±0.028	0.0010	0.953±0.025	< 0.0001
TotalSegmentor	LUL	0.933±0.072	< 0.0001	0.962±0.024	< 0.0001	0.961±0.025	< 0.0001	0.971±0.023	< 0.0001
	LLL	0.906±0.097	< 0.0001	0.958±0.033	< 0.0001	0.953±0.042	< 0.0001	0.959±0.040	< 0.0001
	RUL	0.870±0.098	0.0003	0.951±0.036	<0.0001	0.942±0.037	< 0.0001	0.946±0.033	< 0.0001
	RML	0.848±0.115	0.0021	0.922±0.094	< 0.0001	0.896±0.123	< 0.0001	0.945±0.028	< 0.0001
	RLL	0.853±0.192	0.0003	0.950±0.039	< 0.0001	0.934±0.123	< 0.0001	0.948±0.061	< 0.0001
	Average	0.882±0.055	< 0.0001	0.948±0.028	< 0.0001	0.937±0.024	< 0.0001	0.954±0.022	< 0.0001
LobePrior	LUL	**0.975±0.026**	-	**0.981±0.015**	-	**0.979±0.014**	-	**0.983±0.013**	-
	LLL	**0.961±0.041**	-	**0.975±0.022**	-	**0.974±0.018**	-	**0.976±0.020**	-
	RUL	**0.960±0.030**	-	**0.972±0.023**	-	**0.973±0.012**	-	**0.975±0.016**	-
	RML	**0.930±0.041**	-	**0.958±0.032**	-	**0.958±0.018**	-	**0.964±0.022**	-
	RLL	**0.950±0.038**	-	**0.972±0.017**	-	**0.973±0.017**	-	**0.977±0.017**	-
	Average	**0.955±0.021**	-	**0.972±0.018**	-	**0.971±0.014**	-	**0.975±0.016**	-

Supplementary Table S5. Dice scores and Standard Deviation (STD) values per lung lobe and dataset, with p-values comparing LobePrior versus each method. Bold values indicate the best performance per lobe. Pairwise comparisons were performed using the Wilcoxon signed-rank test. To account for multiple comparisons across lobes and datasets, p-values were adjusted for multiple comparisons using the Holm–Bonferroni method. Therefore, p-values for LobePrior itself are not applicable. The CoronaCases dataset consists of only 10 computed tomography (CT) volumes.
