# Supplementary material for: LobePrior segments lung lobes on computed tomography images in the presence of severe abnormalities
Source: Sci Rep. 2026 Apr 10;16:16205. doi: 10.1038/s41598-026-48136-8 (PMC13201581; doi:10.1038/s41598-026-48136-8)
Supplement: Supplementary file 2 — Supplementary Information 2. [file 41598_2026_48136_MOESM2_ESM.pdf]

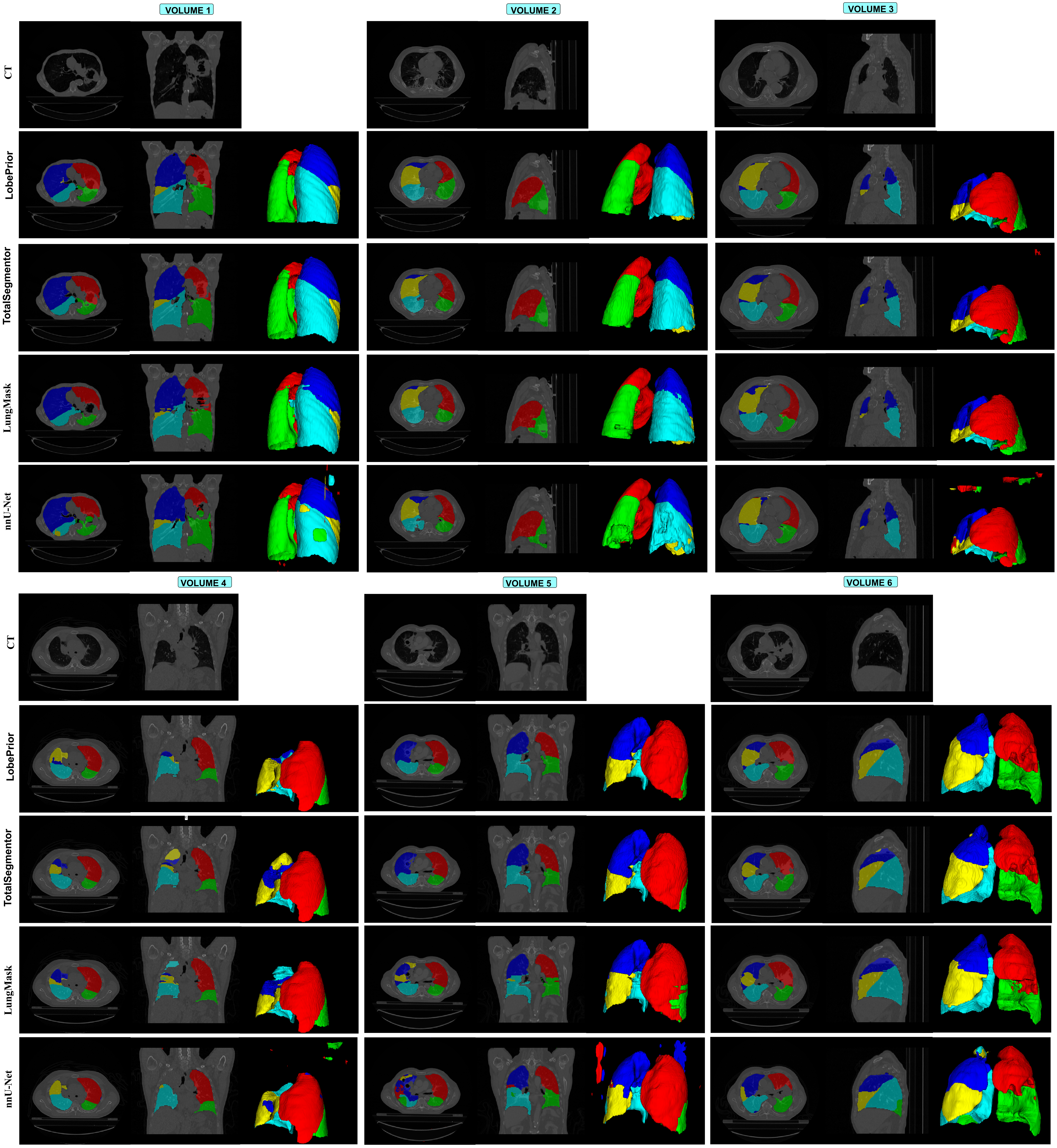

VOLUME 1
VOLUME 2
VOLUME 3
CT
LobePrior
TotalSegmentor
LungMask
nnU-Net
VOLUME 4
VOLUME 5
VOLUME 6
CT
LobePrior
TotalSegmentor
LungMask
nnU-Net
