# Supplementary figures and images for: LobePrior segments lung lobes on computed tomography images in the presence of severe abnormalities
Source: Sci Rep. 2026 Apr 10;16:16205. doi: 10.1038/s41598-026-48136-8 (PMC13201581; doi:10.1038/s41598-026-48136-8)

**A) CT**

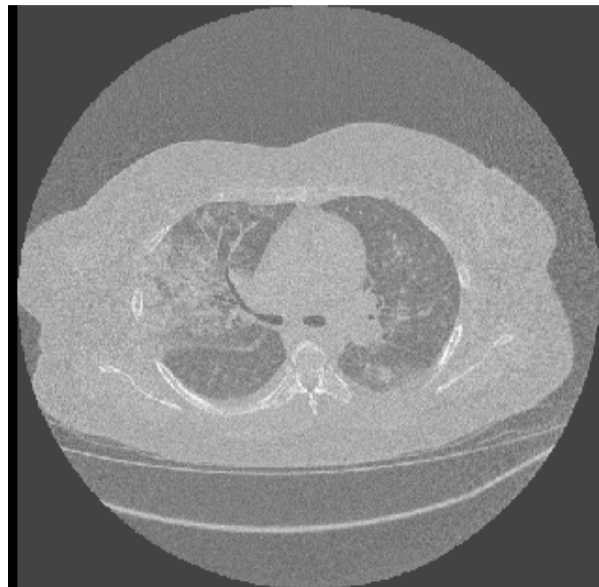

**B) LUNG**

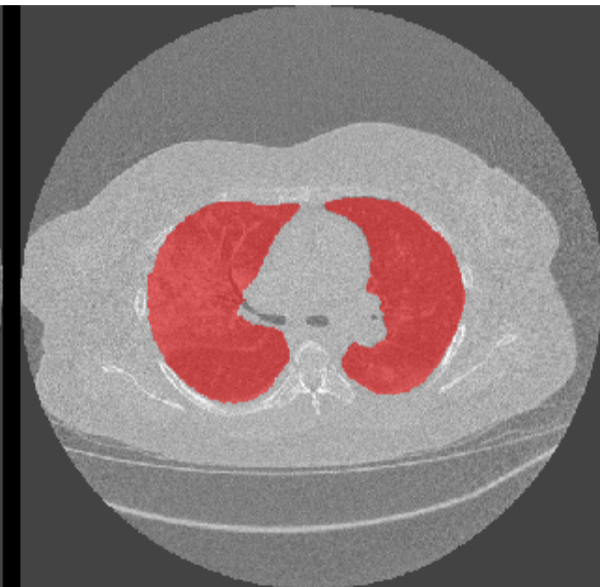

**C) WITHOUT POST**

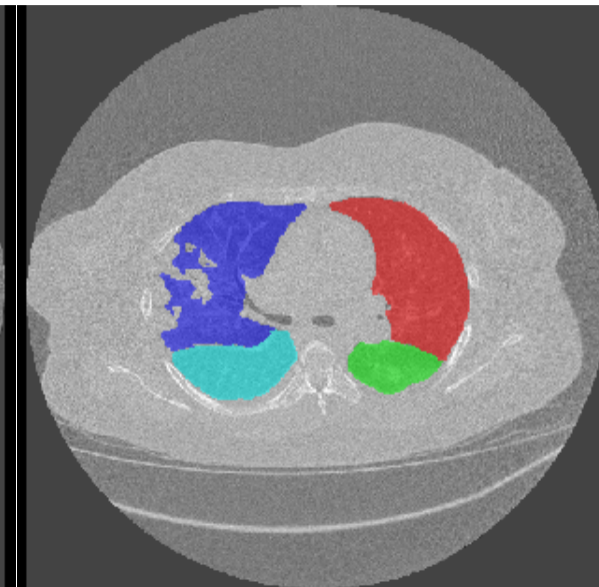

**D) MCC**

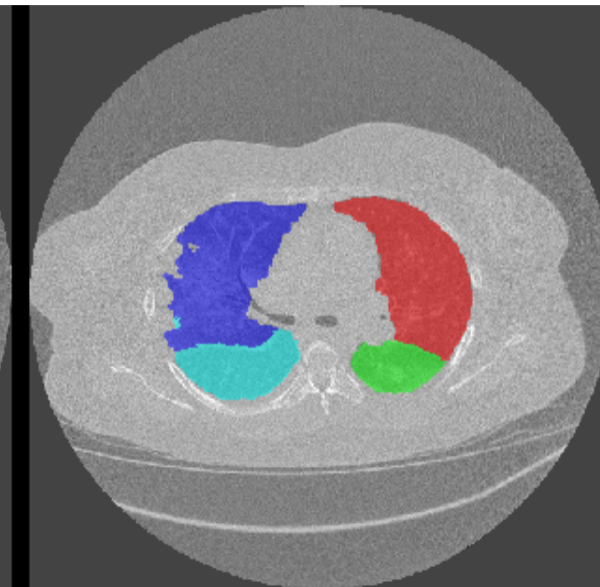

**E) PRIOR**

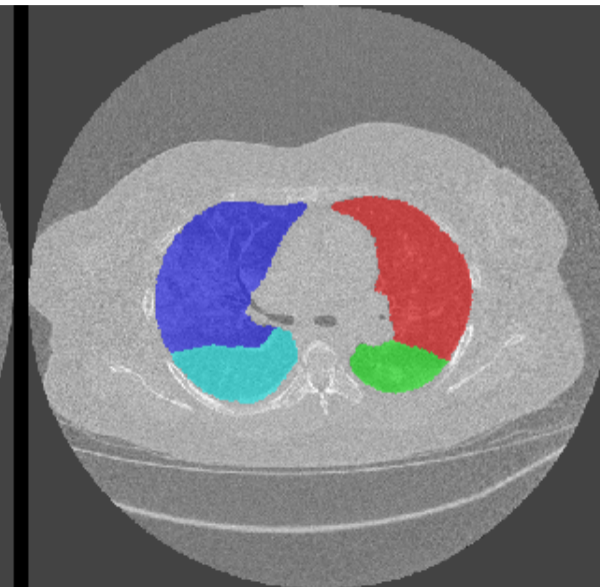

**F) ANNOTATION**

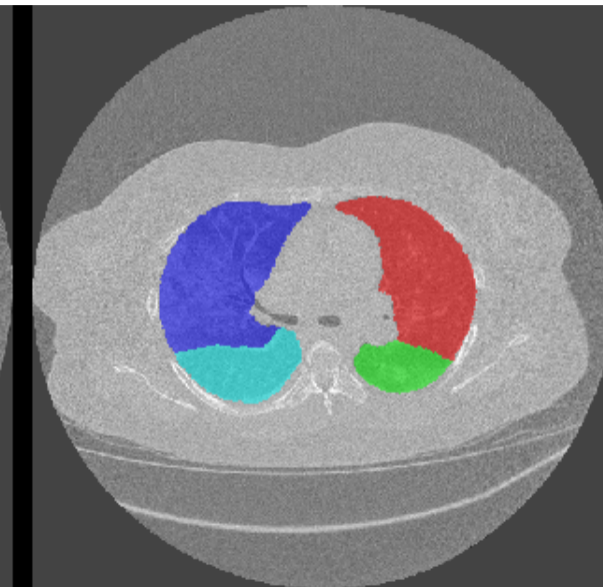

Supplement: Supplementary file 3 — Supplementary Information 3. [file 41598_2026_48136_MOESM3_ESM.pdf]
